# ArgR of *Streptomyces coelicolor* Is a Pleiotropic Transcriptional Regulator: Effect on the Transcriptome, Antibiotic Production, and Differentiation in Liquid Cultures

**DOI:** 10.3389/fmicb.2018.00361

**Published:** 2018-03-01

**Authors:** Alma Botas, Rosario Pérez-Redondo, Antonio Rodríguez-García, Rubén Álvarez-Álvarez, Paula Yagüe, Angel Manteca, Paloma Liras

**Affiliations:** ^1^Área de Microbiología, Departamento de Biología Molecular, Universidad de León, León, Spain; ^2^Instituto de Biotecnología de León, León, Spain; ^3^Área de Microbiología, Departamento de Biología Funcional e IUOPA, Universidad de Oviedo, Oviedo, Spain

**Keywords:** ArgR, arginine, ARG box, *S. coelicolor*, transcriptomics, sporulation, antibiotics

## Abstract

ArgR is a well-characterized transcriptional repressor controlling the expression of arginine and pyrimidine biosynthetic genes in bacteria. In this work, the biological role of *Streptomyces coelicolor* ArgR was analyzed by comparing the transcriptomes of *S. coelicolor* Δ*argR* and its parental strain, *S. coelicolor* M145, at five different times over a 66-h period. The effect of *S. coelicolor* ArgR was more widespread than that of the orthologous protein of *Escherichia coli*, affecting the expression of 1544 genes along the microarray time series. This *S. coelicolor* regulator repressed the expression of arginine and pyrimidine biosynthetic genes, but it also modulated the expression of genes not previously described to be regulated by ArgR: genes involved in nitrogen metabolism and nitrate utilization; the *act, red*, and *cpk* genes for antibiotic production; genes for the synthesis of the osmotic stress protector ectoine; genes related to hydrophobic cover formation and sporulation (chaplins, rodlins, *ramR*, and *whi* genes); all the *cwg* genes encoding proteins for glycan cell wall biosynthesis; and genes involved in gas vesicle formation. Many of these genes contain ARG boxes for ArgR binding. ArgR binding to seven new ARG boxes, located upstream or near the *ectA-ectB, afsS, afsR, glnR*, and *redH* genes, was tested by DNA band-shift assays. These data and those of previously assayed fragments permitted the construction of an improved model of the ArgR binding site. Interestingly, the overexpression of sporulation genes observed in the Δ*argR* mutant in our culture conditions correlated with a sporulation-like process, an uncommon phenotype.

## Introduction

Biosynthesis of amino acids is regulated in microorganisms when these nutrients are abundant in the culture medium. ArgR first described in *Escherichia coli* is the model for the ArgR repressor superfamily; this transcriptional regulator, in response to the presence of arginine, represses the expression of arginine biosynthesis genes using arginine as a co-repressor and decreases the activity of arginine biosynthesis enzymes (Maas, 1994). A similar effect was found for pyrimidine biosynthesis. The ArgR protein is widely distributed in bacteria, acting mostly as a repressor of genes for arginine uptake and biosynthesis (Cunin et al., 1983) but may also act as an activator, as in the *aot* operon for arginine and ornithine uptake in *Pseudomonas* (Nishijyo et al., 1998; Lu et al., 2004). It is an essential accessory protein in the site-specific resolution of ColE1 oligomers in *E. coli* (Stirling et al., 1988).

In gram-positive bacteria, the control of arginine and pyrimidine biosynthesis in *Lactococcus lactis* (Larsen et al., 2008) and the repression of the corynebacteria *argCJBDFR* operon by ArgR are well documented (Yim et al., 2011). L-arginine has been overproduced in a corynebacteria industrial strain by increasing the copy number of the arginine operon genes in an ArgR-defective mutant (Xu et al., 2012). The *Bacillus* AhrC repressor, homologous to ArgR, represses the *argCAEBD*-*cpa*-*argF* gene cluster in the presence of arginine (Smith et al., 1989) and activates arginine catabolism genes in cooperation with the RocR activator (Gardan et al., 1997).

In *Streptomyces coelicolor* and *Streptomyces clavuligerus*, a repression system of the arginine biosynthesis genes homologous to those of other bacteria has been described (Soutar and Baumberg, 1996; Rodríguez-García et al., 1997). The effect of arginine as the ArgR co-repressor is weak in *Streptomyces*, and high levels of this amino acid are required to observe repression of arginine biosynthesis genes or a decrease in arginine biosynthesis enzyme activities (Rodríguez-García et al., 1997). Arginine, when added to *S. coelicolor* cultures at 25 mM, only affected the expression of 27 arginine-related genes (0.35% of the genome; Pérez-Redondo et al., 2012).

The crystallized hexameric ArgR repressor of *E. coli* is formed by two trimers (van Duyne et al., 1996). It has been demonstrated to interact with the operator region of *argF* (Grandori et al., 1995) by binding specific DNA sequences known as ARG boxes. A standard ARG box in *E. coli* is formed by two 18-bp sequences separated by 3 bp and is located close to the promoters of ArgR-controlled genes (Tian et al., 1992). In *E. coli*, ArgR binding to the ARG boxes strictly depends on L-arginine as the co-repressor (van Duyne et al., 1996). In corynebacteria, the ArgR C-terminal end contains a conserved GTIAGDDTL/I oligomerization domain (amino acids 146–154 in *S. coelicolor* ArgR), which has been demonstrated to be essential for arginine binding (Yim et al., 2011). ArgR proteins in *Bacillus* (Dion et al., 1997) show lower specificity and dependency on L-arginine as co-repressor, exhibit an equilibrium trimer-hexamer and bind to ARG boxes normally separated by 2 bp (Song et al., 2002).

DNase I footprinting and electrophoresis mobility shift assay experiments analyzing the binding of *B. subtilis* AhrC to the *argCJBDR* operon of *S. clavuligerus* and the binding of ArgR to the *S. coelicolor* arginine biosynthesis genes (*argC, argG, arcB*, and *argH*) revealed the presence of ARG boxes arranged as two 20-bp contiguous sequences in these actinomycetes (Rodríguez-García et al., 1997; Pérez-Redondo et al., 2012; Botas, 2013), as in the *Bacillus* system (Dion et al., 1997; Song et al., 2002).

Pérez-Redondo et al. (2012) studied the *S. coelicolor* transcriptome at a single developmental time-point (32 h), identifying 459 genes regulated by ArgR. These genes were involved in purine and pyrimidine biosynthesis, cell morphology, and antibiotic production. In this work, we analyzed the differences between the transcriptomes of the parental strain and a Δ*argR* mutant at five different time-points in the culture development, which allowed us to confirm the previous results and significantly increase the number of ArgR-controlled genes at other time points and ratify the significance of data obtained in the single developmental point from previous experiments (Pérez-Redondo et al., 2012). Novel DNA binding experiments enabled the location and characterization of new functional ARG boxes and redefinition of the ARG box model in *S. coelicolor*. A bioinformatic search was performed to locate additional putative ARG boxes that could explain the regulatory role of the ArgR protein. In addition, a sporulation-like phenomenon in liquid culture was observed in the Δ*argR* mutant strain. Sporulation is unusual in *S. coelicolor* and has never been reported for *Streptomyces argR* mutants.

## Materials and methods

### Strains and culture conditions

*S. coelicolor* M145 was used as a control strain (Bentley et al., 2002). *S. coelicolor* Δ*argR* derives from the former strain and is a mutant with a deletion in the *argR* gene (Pérez-Redondo et al., 2012). For transcriptomic studies, *S. coelicolor* strains were inoculated in a defined MG medium containing 50 g/l starch, 12 mM glutamic acid as the only nitrogen source, 2.5 mM phosphate and salts (Doull and Vining, 1989; Pérez-Redondo et al., 2012), using 10^8^ spores stored in glycerol at −80°C. The cultures were grown at 30°C and 300 rpm in triplicate in 500-ml baffled flasks containing 100 ml of medium. Actinorhodin and undecylprodigiosin were spectrophotometrically determined at 640 and 530 nm, respectively, as previously described (Kieser et al., 2000). Dry weight was determined in culture samples (2 ml) washed twice with deionized-ultrapure water and dried for 80 h at 60°C. Growth was similar for both strains (not shown).

### RNA isolation, microarray hybridization, and transcriptomic data analysis

Samples from three independent *S. coelicolor* M145 and *S. coelicolor* Δ*argR* cultures were taken at five time points: 32, 42 (exponential phase start and end) 49, 56, and 66 h (stationary phase). RNA samples with RIN values above 8.5 (2100 Bioanalyzer, Agilent) were employed. Cy5-gDNA and Cy3-cDNA labeling, hybridization in the Sco-Chip^2^-v2 microarrays (Oxford Gene Technology), washing, scanning, and signal quantification were performed as indicated in Yagüe et al. (2014). Fluorescence intensities were processed and normalized using the limma package (Smyth, 2004) in the R environment as indicated previously (Yagüe et al., 2014), except that quality weights were estimated for the non-control spots (43,798) of each array (Table S1). These weights were used for normalization and linear model statistics. For each gene, the normalized Mg values were calculated as the average among three replicates of the binary logarithms of the Cy3-cDNA signal divided by the Cy5-gDNA signal (log Cy3/Cy5). Probe values were previously averaged if more than one probe for a gene were present (mean of 4.6 probes per gene in the arrays). For each culture time, comparisons between the Mg values of the mutant and parental strains were the basis of the statistical results and corresponded to the Mc value (fold change). A threshold of 0.01 for the Benjamini-Hochberg adjusted *p*-values was used to identify significantly differentially expressed genes. This resulted in 1544 genes (out of a total of 7,721 present in the microarray), which passed the threshold in at least one comparison. Significantly differential expression profiles where identified in the time-course microarray experiment by means of maSigPro (Conesa et al., 2006) and grouped in 10 profiles. The GEO accession number for microarray data is GSE58666.

### qRT-PCR

The qRT-PCR was performed in triplicate RNA samples with the oligonucleotides shown in Table S2, as previously described (López-García et al., 2010). RNA was retrotranscribed to cDNA using random primers and the Invitrogen SuperScript III commercial kit. Amplification and quantification of DNA by qRT-PCR was performed using a StepOnePlus thermocycler using SYBR Green PCR Master Mix (both from Applied Biosystems). The baseline and threshold cycle determination was performed using Sequence Detection Software (Applied Biosystems).

The reactions were prepared in a final volume of 20 μl. A template of 2 μl of undiluted (or 2- to 10-fold diluted) cDNA was used to give Ct detection between cycles 15 and 25. The final concentration of 300 μM oligonucleotides increased the highest amplification of the specific product at a lower Ct without primer dimer formation. The RNA was confirmed to be free of DNA contamination using negative controls where template cDNA was replaced with RNA.

The relative quantification of the expression differences of a target gene between mutant and control strains was performed using the ΔΔCt method (Livak and Schmittgen, 2001). As a reference, the *hrdB* gene, encoding a constitutive *Streptomyces* sigma factor (Buttner et al., 1990), was used.

The efficiency of each oligonucleotide pair was determined by amplifying serial dilutions of genomic DNA (six different dilutions, each amplified in triplicate) and measuring the slope of the resulting line of Ct plotted against the logarithm of DNA concentration. Slope values between −3.6 and −3.1 were regarded as valid, indicating efficiencies of 90–100%, which were required to apply the ΔΔCT method.

The relative expression of a gene in the mutant strain is given by 2^−ΔΔCt^, where ΔΔCt indicates the difference between the ΔCt of both strains analyzed, obtained from the difference in the Ct of the target gene and the reference gene in each strain. Relative expression above 1 indicated that the analyzed gene is overexpressed in the mutant strain, while values below 1 indicate its repression.

### DNA band-shift studies and structure of the ArgR binding site

To improve the previous model of the ArgR binding site (Pérez-Redondo et al., 2012), we used the DNA band-shift assays (EMSA, electrophoretic mobility shift assay) results for 50 DNA fragments. The conditions used were as indicated in a previous work (Pérez-Redondo et al., 2012).

In brief, ArgR protein was purified from *E. coli* as a Strep-tag fused protein. DNA fragments to be tested by EMSA were amplified using specific oligonucleotides (Table S2), cloned in pBluescript SK+ (Stratagene), and labeled by PCR with Universal 6-FAM oligonucleotides to obtain fluorescent probes. The DNA-protein binding reaction contained: 5 μL buffer (10 mM Tris–HCl, pH 7.4, 5 mM MgCl2, 2.5 mM CaCl2, 250 mM KCl, 0.5 mM DTT, 10 mM L-arginine, pH 7.4), poly-(dIdC) 1,3 μg/mL, glycerol 10%, 6-FAM-labeled probe 2 nM, and Strep-ArgR protein 0.8 μM in 15 μL. This mixture was incubated for 30 min at 30°C and immediately resolved in a 5% acrylamide gel using 0.5x TBE as running buffer at 50 V. Competition experiments were done with increasing amounts of unlabeled specific probe. The chromosomal sequences of the probes used for DNA binding shift are included in Table S3.

To improve the previous model of the ArgR binding site (Pérez-Redondo et al., 2012), we used the EMSA which resulted in 50 DNA fragments shifted. The chromosomal sequences of the probes used for DNA band-shift assays, obtained by PCR amplification, are included in Table S3. These sequences were chosen among those containing putative ArgR binding sites, according to the previous model. A three-step process was conducted to identify ARG boxes in the sequences of the positive probes and to build a new binding-site model. First, the MEME algorithm, available through the MEME server (Bailey et al., 2009), was used for motif discovery using two search strategies: (i) the discriminative mode, fed with both sets of positive and negative sequences and searching for palindromes 14–20 nt in length (ZOOPS option), detected a total of 25 ARG boxes in the input positive sequences and built an ARG box model 14 nt in length (named ARGNE04); (ii) the normal mode, searching for palindromes 18–20 nt in length with the ANR option, detected 14 sites among the set of positive probes and produced a model 20 nt in length (ARGNE05). Second, the sequences giving positive DNA band-shift were scanned with both the ARGNE04 and ARGNE05 models using the FIMO algorithm (Grant et al., 2011). The results were manually inspected to determine the most likely binding site(s) in each positive sequence, in terms of sequence conservation, location relative to the translation start site of the regulated gene, and the presence of a unique ARG box or two tandemly arranged ARG boxes (Table 1). Third, 37 ARG boxes were well-conserved sequences that were selected among the 44 boxes identified in the previous analysis and used to build the final model using information theory algorithms (Schneider, 1997).

**Table 1:** ARG boxes detected in DNA fragments bound by ArgR in DNA band-shift assays.

					**Chromosomal coordinates**			
**Regulated gene(s)**	**Gene name**	**Product**	**ARG box identifier**	**Sequence**	**Left**	**Right**	**Distance to start codon**	**R*i* (bit)**
SCO0256 ^a^^,^^f^		Short chain oxidoreductase-like	AB.SCO0256_1	GCCCGCACGCTGTTGGGCAG	245912	245931	55	5.8
SCO0256 ^a^^,^^f^		Short chain oxidoreductase-like	AB.SCO0256_2	CACCGCACGAGAGCGCGGAG	245932	245951	35	7.7
SCO1086 ^a^		Hypothetical protein	AB.SCO1086_1	CAGTCAATGACTTTTCAGGT	1146431	1146450	5	5.0
SCO1086		Hypothetical protein	AB.SCO1086_2	GAACGCATACCTATGCAGTG	1146451	1146470	25	15.7
SCO1236	*ureA*	Urease gamma subunit	AB.SCO1236_0	GATTGATTACCGATGCAGTC	1310778	1310797	183	7.4
SCO1483 ^c^	*pyrA*	Carbamoylphosphate synthetase L chain	AB.SCO1483_0	GGTCGAACAGGTAGGCGGCG	1587621	1587640	209	5.6
SCO1487	*pyrB*	Aspartate carbamoyltransferase	AB.SCO1487_0	CTCCGTAAGGCGATTCATGC	1591396	1591415	9	9.3
SCO1488	*pyrR*	Pyrimidine operon regulatory protein	AB.SCO1488_0	TGTTGCTTGTCCATACGAAA	1592070	1592089	−13	7.4
SCO1489	*bldD*	BldD, transcriptional regulator	AB.SCO1489_0	CGCTGCGTAACCTCACAGTG	1592299	1592318	63	9.6
SCO1570 ^a^	*argH*	Argininosuccinate lyase	AB.SCO1570_1	TCATGCATGAGTATGCAGAA	1681889	1681908	30	16.3
SCO1570	*argH*	Argininosuccinate lyase	AB.SCO1570_2	CTCTGCATGTTTCGTCAATC	1681909	1681928	50	4.4
SCO1580 ^c^	*argC*	N-acetyl-γ-glutamyl-phosphate reductase	AB.SCO1580_A0	GCCCGCATACCCACTCGCTC	1691417	1691436	−44	9.5
SCO1580 ^a^^,^^d^	*argC*	N-acetyll-γ-glutamyl-phosphate reductase	AB.SCO1580_B1	GAGAGCATGACTATACGTGC	1691479	1691498	18|-3	9.1
SCO1580 ^d^	*argC*	N-acetyl–γ-glutamyl-phosphate reductase	AB.SCO1580_B2	CGATGCACGTTTATGCAATG	1691499	1691518	38|-23	13.3
SCO1864 ^c^^,^^e^		Putative acetyltransferase	AB.SCO1864_0	ATTCGTAACCCCATCCGGCG	1998411	1998430	38	3.8
SCO2015		2′,3′-cyclic-nt 2′-phosphodiesterase-like	AB.SCO2015_0	CCACGCATGACCACTCAACA	2157812	2157831	159	11.4
SCO2055 ^b^		Hypothetical protein	AB.SCO2055_1	GTTCGGACGGTCACGCAGTG	2202678	2202697	63	11.8
SCO2055		Hypothetical protein	AB.SCO2055_2	GCATGAACGGTTGTACGAAG	2202699	2202718	42	9.0
SCO2231-2 ^b^	*malE-R*	Maltose-binding, transcriptional repressor	AB.SCO2231-2_A1	TGCTGCAAAAATGTGCAAGA	2400395	2400414	136|237	10.7
SCO2231-2	*malE-R*	Maltose-binding, transcriptional repressor	AB.SCO2231-2_A2	ATCTCCGAAGTTATCCGGGC	2400416	2400435	157|216	5.1
SCO2231-2	*malE-R*	Maltose-binding, transcriptional repressor	AB.SCO2231-2_B0	CTCTGCAAGCTCTTGCCGCC	2400507	2400526	248|125	11.9
SCO2529	*smpA*	Metalloprotease	AB.SCO2529_0	GTCCGTATCAGTATGCGGGA	2727401	2727420	144	10.7
SCO2686 ^b^^,^^c^^,^^f^		Putative luxR-family transcriptional regulator	AB.SCO2686_A1	TATTGAGCGTCCTTTCAAAA	2930741	2930760	−98	5.5
SCO2686 ^c^^,^^f^		Putative luxR-family transcriptional regulator	AB.SCO2686_A2	TCGCGCATAGAAGTGCAGTC	2930762	2930781	−77	7.3
SCO2686 ^f^		Putative luxR-family transcriptional regulator	AB.SCO2686_B0	CAATGCATGATCATGCCACA	2930862	2930881	23	12.6
SCO3034^f^	*whiB*	Sporulation regulatory protein	AB.SCO3034_0	GGAACCAAGCCCATGCAGTA	3321156	3321175	140	9.4
SCO3067 ^f^	*arsI|sigI*	Anti anti sigma factor|sigma factor SigI	AB.SCO3067_0	CACAGAACACACTTGCGGTC	3360549	3360568	108|126	7.6
SCO3943 ^b^^,^^f^	*rstP*	Putative transcriptional regulator	AB.SCO3943_A0	GGACGCATATGCACGCGTTG	4339185	4339204	39	10.2
SCO3943 ^b^^,^^f^	*rstP*	Putative transcriptional regulator	AB.SCO3943_B0	TTCTGCAAGATCATTAATGC	4339215	4339234	69	9.4
SCO3978-9 ^f^		Oxidoreductase|TetR-like regulator	AB.SCO3978-9_0	TAACGGATAGCTTTTCATAA	4381976	4381995	27|35	9.2
SCO4158^f^		LacI-family regulatory protein-like	AB.SCO4158_0	CCTTGGATGACCTTGCGCCC	4576182	4576201	48	11.3
SCO4293^f^		Putative threonine synthase	AB.SCO4293_0	GCCTCCATGGCTGTGCAGAC	4708595	4708614	−7	14.5
SCO4425	*afsS*	Sigma-like protein	AB.SCO4425_0	TGCCGGACGGGCGCGCGGAG	4842637	4842656	64	8.8
SCO4426 ^c^^,^^f^	*afsR*	Regulatory protein	AB.SCO4426_0	TTTGCCTTGTTCATGCCGAC	4845804	4845823	36	1.2
SCO5226^f^	*nrdA*	Ribonucleotide-diphosphate reductase	AB.SCO5226_0	GACTGGACAGGCGTGCGCGC	5688301	5688320	158	9.2
SCO5326-7^f^		Hypothetical protein	AB.SCO5326-7_0	GCCTCGTTGGTCATGCATCC	5796724	5796743	146|-7	8.9
SCO5583	*amtB*	Ammonium transporter	AB.SCO5583_0	CCATGCCAGGTCATTCGGAG	6085777	6085796	235	10.2
SCO5864^f^		Conserved hypothetical protein	AB.SCO5864_0	CTCTCCGTGATCATGCACCC	6421332	6421351	267	11.8
SCO5896 ^c^	*redH*	Phosphoenolpyruvate-utilizing enzyme	AB.SCO5896_0	GACGGCGTGGGCCTGCAGAA	6461067	6461086	−1669	6.3
SCO5976 ^a^	*arcB*	Ornithine carbamoyltransferase	AB.SCO5976_1	CGCTGTATAGAAATTCAGAA	6550325	6550344	55	8.2
SCO5976	*arcB*	Ornithine carbamoyltransferase	AB.SCO5976_2	GTTCGTATAGACTTCCAGAA	6550345	6550364	35	9.6
SCO7036 ^a^	*argG*	Argininosuccinate synthase	AB.SCO7036_1	CTTTGCATGGTCATGCGTAA	7824734	7824753	18	17.2
SCO7036	*argG*	Argininosuccinate synthase	AB.SCO7036_2	TGATGCATACTCTTCCTATG	7824754	7824773	−2	8.1
SCO7314	*sigM*	Probable RNA polymerase sigma factor	AB.SCO7314_0	ATCCGCATGCTCATAGAAAC	8120535	8120554	−13	9.5

a , No separation between the ARG boxes;

b , One nucleotide separation between ARG boxes;

c , Not included in the model;

d , Binding site “A” might to control both SCO1580 and SCO1581 genes;

e , Alternative box located 27 nt before;

f *, Differential transcription not observed*.

### Viability stain

Culture samples were obtained and processed for microscopy at different incubation time-points, as previously described (Manteca et al., 2007, 2008). To detect the dead cell population, the cells were stained with the cell-impermeant nucleic acid stain propidium iodide (PI), which only penetrates bacteria with damaged membranes. In addition, SYTO 9 green fluorescent nucleic acid stain, which labels all cells (LIVE/DEAD BacLight Bacterial Viability Kit, Invitrogen) was used to detect viable cells. In the presence of both stains, bacteria with intact cell membranes appeared to fluoresce green, whereas bacteria with damaged membranes appear red. After being left to sit at least 10 min in the dark, the samples were examined under a Leica TCS-SP2-AOBS confocal laser-scanning microscope at a wavelength of either 488 or 568 nm excitation and 530 nm (green) or 630 nm (red) emission, respectively (optical sections ~0.2 μm). Images were mixed using Leica Confocal Software. In some cases, samples were also examined in differential interference contrast mode using the same equipment.

Images were processed with ImageJ. Compartmentalized hyphae were counted using the cell counter plugin (https://imagej.nih.gov/ij/plugins/cell-counter.html). The percentage of hyphae suffering segmentation (sporulation-like) was estimated by counting 727 hyphae among numerous pictures, and two different biological replicates, visualized independently in the same focal plane. The average segment length was estimated from 226 measurements (Figure S1).

## Results

### Construction of a new model to analyse ArgR binding in *S. coelicolor*

**Figure 1:**
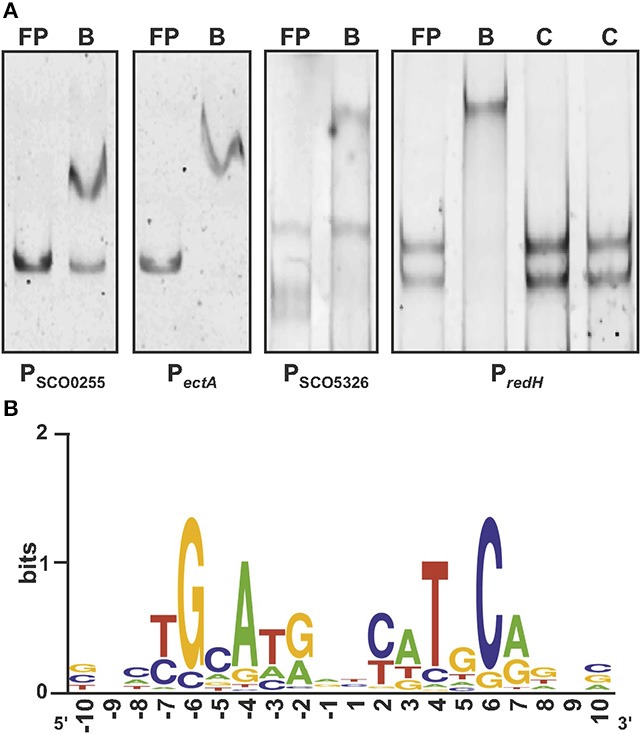
DNA band-shift assays of new ARG boxes and Sequence Logo. **(A)** ArgR binding analysis of DNA fragments containing ARG boxes. Free probe (FP), binding reaction (B), competition reactions with non labeled probe (C). In all cases competition reactions were made to determine the binding specificity, however it is only shown the competition for the *redH* binding assay (amount of competitor in the reactions: 9.5 and 19x, left and right, respectively). Assays were performed on intergenic regions of SCO0255*-*SCO0256 and SCO1863-1864 (*ectA*) and coding regions of SCO5326 and SCO5896 (*redH*). **(B)** Sequence logo of ARGNE06 model. Letter height is proportional to the base frequency in aligned sequences used to build the model, and letter stack height is conservation in bits at that position.

Previous footprinting, EMSA and *in vivo* luciferase-fused sequence data demonstrated ArgR binding to several gene promoters (Rodríguez-García et al., 1997; Pérez-Redondo et al., 2012). ArgR binding sites (ARG boxes) are imperfect palindromes up to 20 nt in length (two turns of the DNA helix). Most evident ArgR binding sites were identified in the arginine biosynthesis promoters of *S. clavuligerus* (Rodríguez-García et al., 1997) and *S. coelicolor* (Pérez-Redondo et al., 2012). All binding sites are composed of two contiguous ARG boxes, although DNA band-shift studies showed ArgR binding sites, formed by a unique ARG box (Pérez-Redondo et al., 2012). A bioinformatics model of the *S. coelicolor* ARG box was built according to these sequences (Pérez-Redondo et al., 2012). In this work, the results of EMSA with 50 DNA fragments (27 previously published) were used to build an improved model of the ArgR binding site. Of these 50 fragments assayed, 30 yielded mobility shifts, and 20 fragments failed to show ArgR binding (Table S3). Seven novel positive fragments correspond to the intergenic regions of SCO0255-SCO0256 and SCO1863-SCO1864 (*ectA*-*ectB*) genes, to the upstream regions of SCO4425 (*afsS*) and SCO4426 (*afsR*) genes, and to the coding regions of SCO4159 (*glnR*), SCO5326 and SCO5896 (*redH*). All the experimental data described in Materials and Methods allowed that a new model of the ARG box was built (Figure 1). The total conservation of this model is R*sequence* = 9.9 bits, and the R*i* value of the consensus sequence is 20.9 bits.

The new model was used to analyse the ArgR binding sites present in the DNA fragments giving positive EMSA. A total of 44 ARG functional boxes were identified, showing various conservation values (Table 1). These ARG boxes were arranged into three types of binding sites: (1) typical binding sites formed by two contiguous ARG boxes, such as those of *arg* genes; (2) binding sites formed by two tandem ARG boxes but separated by one nucleotide; and (3) binding sites formed by a single ARG box. In the promoter of the *rstP* gene, there are two possible ARG boxes separated by 10 nucleotides. It is possible that both boxes form a single binding site or constitute two independent sites.

To identify ARG boxes in the genes transcriptionally affected by the lack of ArgR (see below), a bioinformatic search was conducted in the *S. coelicolor* chromosome. The list of predicted ArgR binding sites was filtered by probability (*p*-value < 10^−5^) and information content (R*i* > 10.0 bit) (Table S4A). The 315 ArgR putative sites shown control 221 differentially expressed genes, at either the gene located downstream of the ARG site or the next. In addition, many ArgR binding sites with lower probability were identified, some of which were related to differentially transcribed genes; some are shown in Table S4B. The functionality of most of these ARG boxes remains to be validated.

### Transcriptomic studies of *S. coelicolor* M145 and *S. coelicolor* Δ*argR*

**Figure 2:**
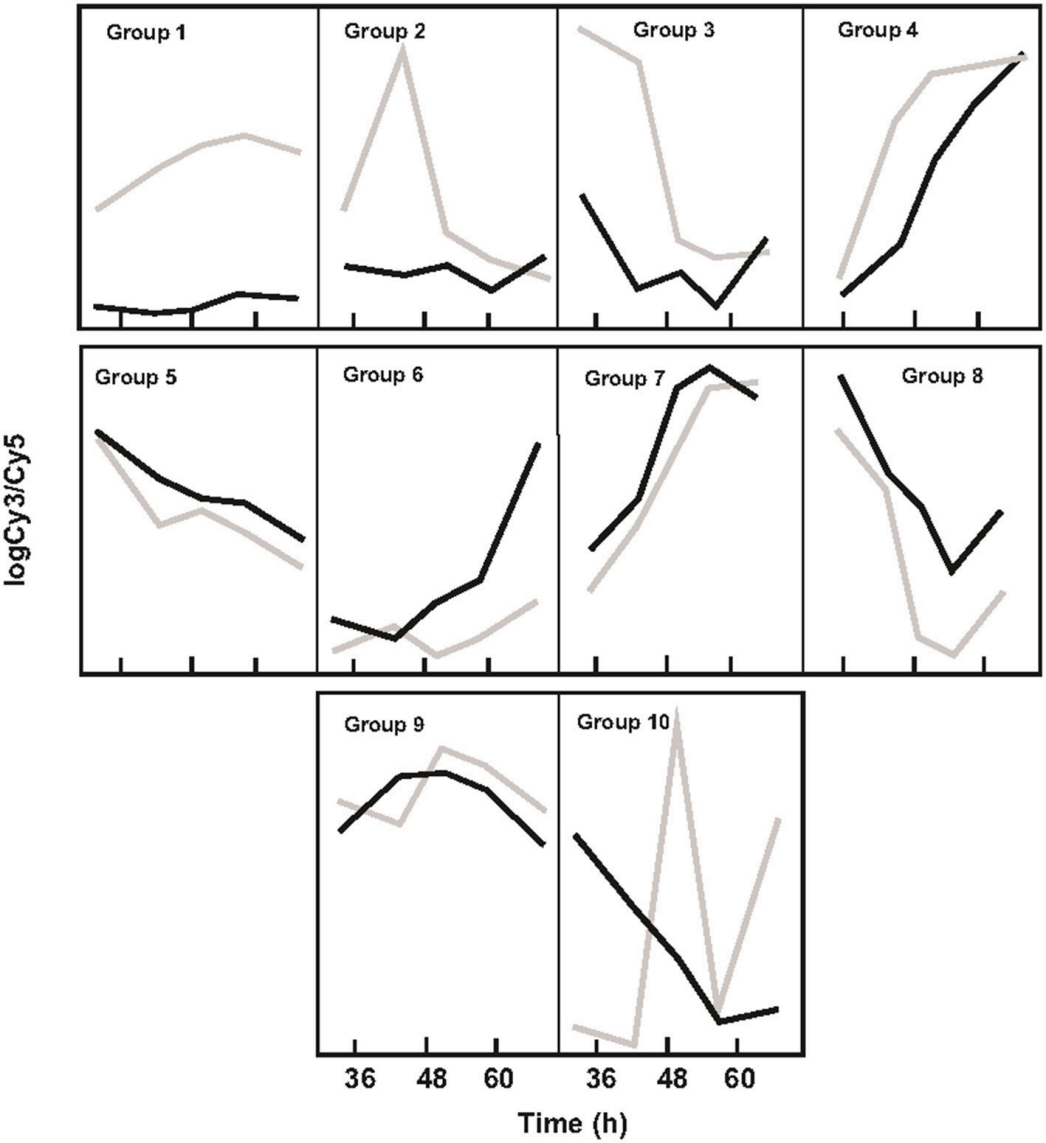
Clustering of gene expression profiles in *S. coelicolor* M145 and *S. coelicolor* Δ*argR*. *S. coelicolor* M145 gene expression is indicated by black lines; expression of *S. coelicolor* Δ*argR* gene is indicated by gray lines. Clustering was obtained using maSigPro Program.

Gene expression was analyzed in MG liquid cultures of *S. coelicolor* M145 and *S. coelicolor* Δ*argR*, employing three biological replicates at five time points. A total of 1544 genes (~20% of the *S. coelicolor* genome) showed differences in expression (signification level *p* < 0.01) in at least one of the 5 time points analyzed. These transcripts corresponded to the genes involved in amino acid metabolism (75 genes), purine and pyrimidine metabolism (31 genes), nitrogen and phosphate control (20 genes), DNA repair and recombination (25 genes), structure and morphology (78 genes), secondary metabolism (74 genes), coenzyme biosynthesis (13 genes), two-component systems (57 genes), regulators and sigma factors (106 genes), or membrane protein-encoding genes (160 genes). Many genes were related to protein secretion or had unknown functions (767 genes), and others were unclassified genes (138) (Table S5). The differentially expressed genes in the control strain and Δ*argR* mutant were fitted into ten prototypical expression patterns (Figure 2). ArgR behaves mainly as a repressor (profiles 1, 2, 3, and 4) but can also be a weak activator (profiles 5, 6, 7, and 8), and few genes showed either repression or activation at various growth times (profiles 9 and 10). It has to be noted that 50% of the genes did not fit any of the 10 maSigPro profiles. Only 45 of the 7,721 genes scanned were deregulated at all times.

### Genes related to amino acids and pyrimidine biosynthesis

**Figure 3:**
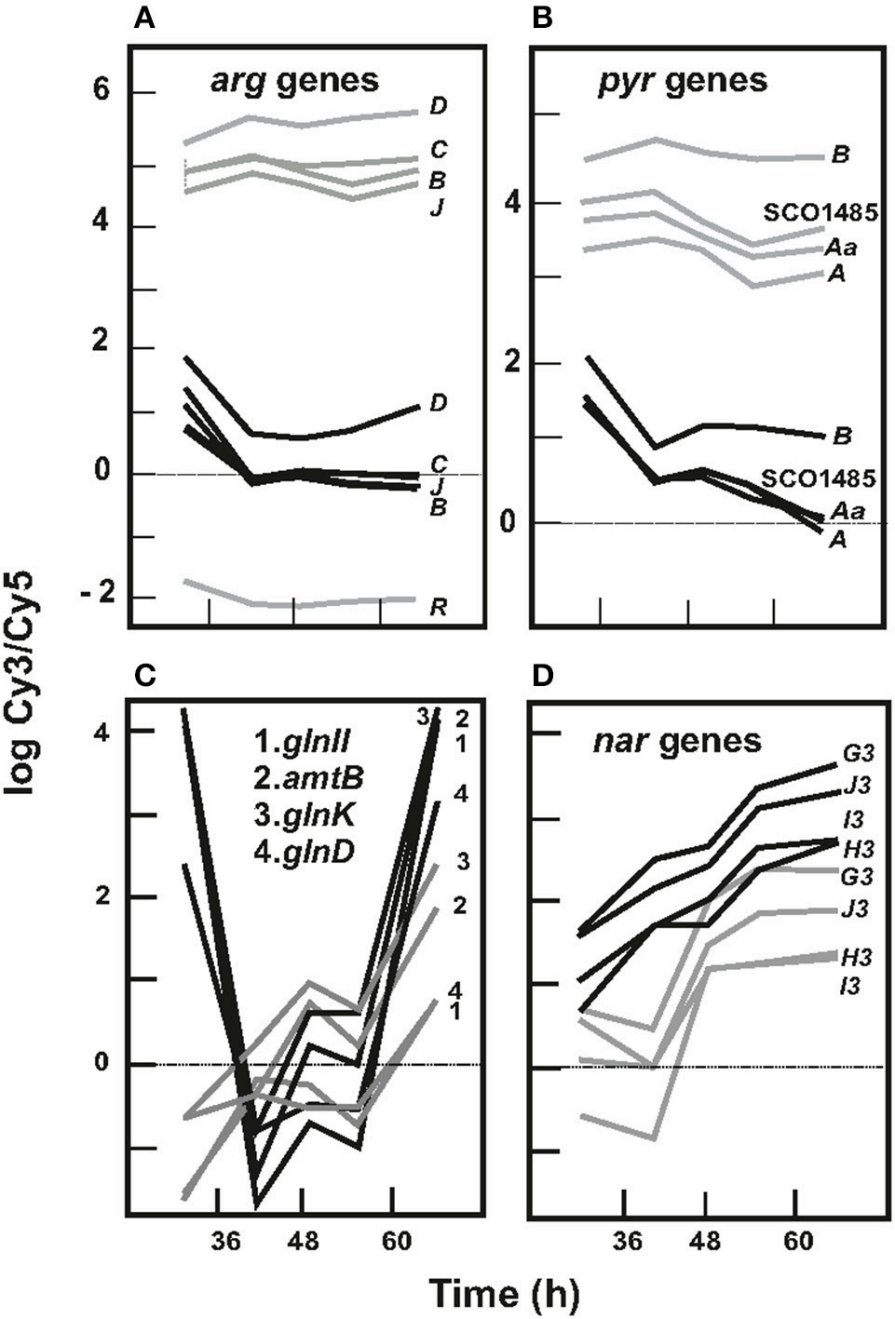
Expression profile of different genes in *S. coelicolor* M145 and *S. coelicolor* Δ*argR*. **(A)** Arginine biosynthesis genes. Profiles of *argB, argC, argD, argJ*, and *arg*R are shown. Mg values for *argR* probe in mutant strain correspond to a nonexistent *argR* gene, and, thus serve to assess Mg values reflecting lack of expression. **(B)** Pyrimidine biosynthesis genes. Only *pyrAa, pyrA, pyrB*, and SCO1485 are shown as a model. **(C)** Profile of *glnII amtB, glnK*, and *glnD* as model of nitrogen metabolism genes. **(D)** Expression profile of *nar3* genes. *S. coelicolor* M145 genes (black lines) and *S. coelicolor* Δ*argR* genes (gray lines).

The genes most affected by the absence of ArgR were those involved in arginine biosynthesis (Figure 3A). They were overexpressed in the Δ*argR* mutant, in agreement with previous observations for type II ArgR repressors (Tian et al., 1992). Fold changes (or Mc values) for these transcripts oscillated between 7- and 38-fold upregulation in the mutant, and *argC* was the most upregulated gene (Table S5). Conserved ARG boxes were located upstream of *argC, argH, argR, arcB*, and in the intergenic *argG-gabD* bidirectional promoter region (Pérez-Redondo et al., 2012; Table 1). Amino acid biosynthesis genes, such as *hppD* (SCO2927) and *glyA3* (SCO5364) involved in glycine-serine-threonine metabolism or *gabD* (SCO7035) encoding a succinate semialdehyde dehydrogenase, were upregulated in the Δ*argR* mutant (Table S5). Especially remarkable was the effect on gene SCO1086, encoding a protein with a transglutaminase domain (125-fold upregulation) (Table S5).

Genes for pyrimidine biosynthesis were highly upregulated in the Δ*argR* strain, with fold changes close to 4.0; *pyrB* and *pyrR* were the most upregulated genes (Figure 3B; Table S5). Conserved ARG boxes are present upstream of *pyrB, pyrA, pyrD*, and *pyrR* (Table 1), and some were already confirmed to be bound by ArgR *in vitro* (Pérez-Redondo et al., 2012). The ribonucleotide reductases, forming deoxyribonucleotides in an oxygen-dependent (*nrdABS*) or oxygen-independent (*nrdRJ*) manner, were upregulated by the absence of ArgR but only at the early exponential growth phase (32 h) (Pérez-Redondo et al., 2012). The same was true for the *cobB* and *cobQ* genes required for cobalamin B12 formation, a cofactor controlling *nrdABS* transcription (Table S5).

### Genes related to nitrogen metabolism

The expression of nitrogen metabolism genes (*glnII, glnA, amtB, glnK*, and *glnD*) in *S. coelicolor* M145 is shown in Figure 3C. These genes showed high expression at early times, moderate expression between 42 and 56 h, and another increase at the end of the culture. This pattern does not clearly fit any of the profiles shown in Figure 2. The Δ*argR* mutant displayed expression similar to the control strain between 42 and 56 h of growth, but the strong upregulation at early and late times observed in the control strain did not occur in the mutant (Figure 3C).

*S. coelicolor* grows on nitrate as sole nitrogen source, and it possesses three gene clusters for nitrate reduction: SCO0216 to SCO0219 and SCO4947 to SCO4750, complexes 2 and 3, respectively, and SCO6532 to SCO6535 (Fischer et al., 2010). All genes encoding for the nitrate reductase complex 3 are underexpressed in the Δ*argR* mutant (Figure 3D), with an expression profile that fitted in group 6, as shown in Figure 2. Functional ARG boxes are present upstream of *amtB* (Pérez-Redondo et al., 2012) and in the 3′ region of SCO4159, *glnR* (Table 1).

### Membrane and secretion proteins

Approximately 150 genes encoding proteins related to secretion and 160 genes for membrane proteins were up- or down-regulated in expression compared to the parental and the Δ*argR* strains (Table S5). Some of these genes were strongly underexpressed (SCO4251 and SCO6934) in the mutant at early or late culture times. Other genes, especially SCO0615, SCO0665, SCO6375, and SCO2704, are overexpressed in the Δ*argR* mutant (Table S5).

### Secondary metabolism gene clusters

Lack of ArgR affects the production of the pigmented antibiotics actinorhodin and undecylprodigiosin (Pérez-Redondo et al., 2012). Red and Act production in the control strain reached 6 and 50 nmol/mg dry weight and were detected at 56 and 66 h, respectively. The mutant antibiotic production was reduced to 12% (for Act) and 8% (for Red) of the levels detected in the parental strain (not shown).

**Figure 4:**
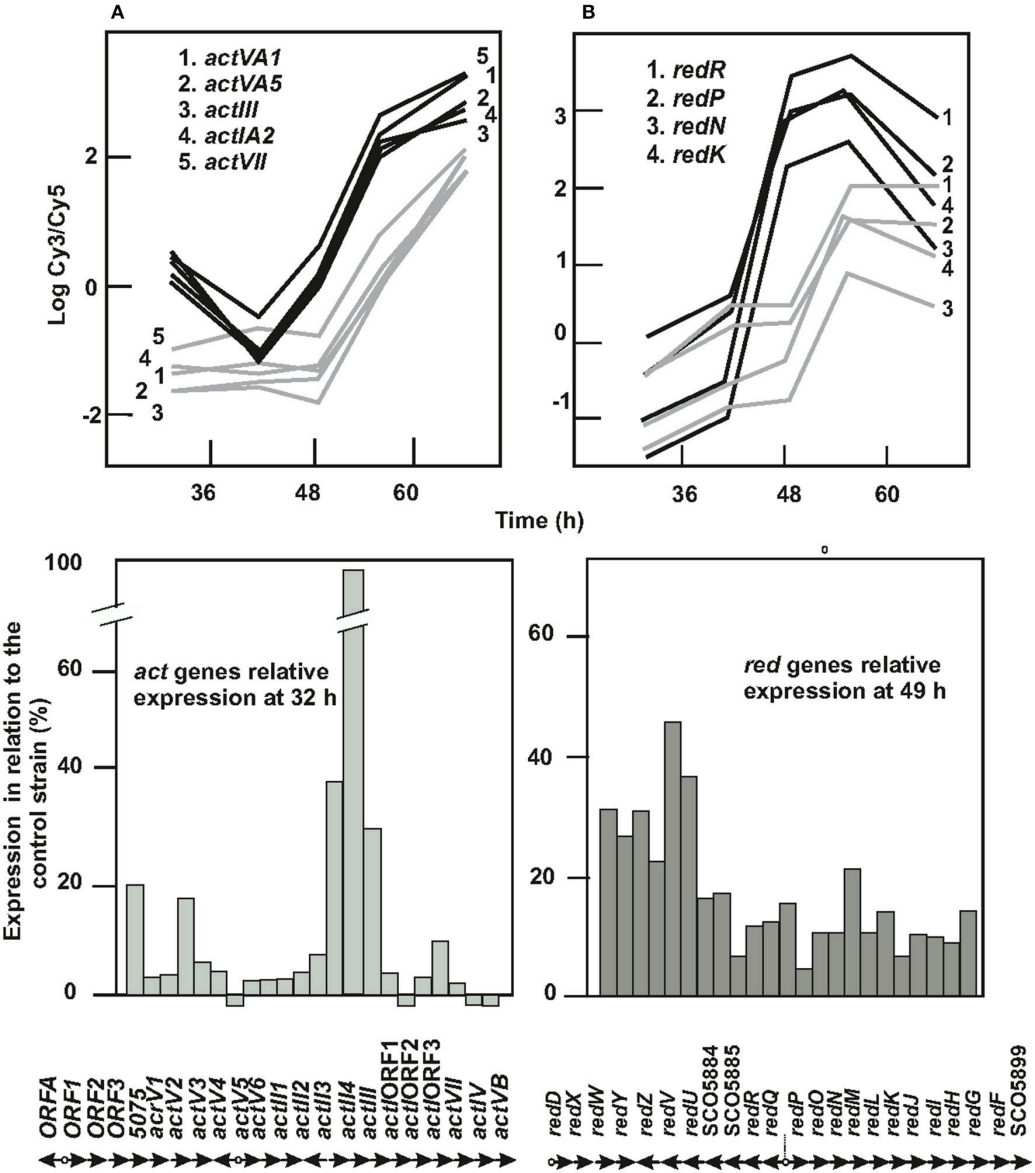
Expression of *act* and *red* genes in *S. coelicolor* M145 and *S. coelicolor* Δ*argR*. **(A)** Actinorhodin biosynthesis genes. Expression profile of *actVA1, actVA5, actII, actIA2*, and *actVII* are shown as model (upper left panel). In lower left panel, columns show the relative expression of each *act* gene in *S. coelicolor* Δ*argR* at 32 h compared to *S. coelicolor* M145 expression, taken as 100. The corresponding genes are indicated below. **(B)** Undecylprodigiosin biosynthesis genes. Expression profile of *redR, redP, redN*, and *redK* are shown as a model (upper right panel). In lower right panel columns show relative expression of each *red* gene in *S. coelicolor* Δ*argR* at 49 h compared to *S. coelicolor* M145 expression, taken as 100. The corresponding *red* genes are indicated below*. S. coelicolor* M145 genes (black lines), *S. coelicolor* Δ*argR* genes (gray lines). The time point at which the expression difference between strains of both sets of genes is maximal (32 or 49 h) have been chosen for representation in lower panels.

All genes for actinorhodin biosynthesis (SCO5071 to SCO5092) shared the same expression profile with growth (group 6 in Figure 2). Expression of the *act* genes in the parental strain decreased from 32 to 42 h and increased steadily thereafter to reach a 6-fold level, whereas the genes expression in the mutant was always lower (18–55% of the level of the parental strain) and increased after 49 h to reach a final level 23% lower than that of the parental strain (Figure 4A).

The 23 genes involved in undecylprodigiosin biosynthesis (SCO5877 to SCO5899) were repressed in the Δ*argR* mutant with respect to the control strain (Figure 4B), following a group 7 profile. As shown below, the coding sequence of *redH*, SCO5896, contains a functional ARG box (6.3 bits, Table 1).

Expression of the *cpk* gene cluster (SCO6268 to SCO6288) for the biosynthesis of the polyketide coelimycin P1 (Gomez-Escribano et al., 2012) was also altered in the Δ*argR* mutant. The *cpk* genes' expression decreased steadily in the control strain with time, while in the Δ*argR* mutant, the transcription increased to a maximum at the 49 h sampling time and then decreased. However, the complex transcription profile of these genes (group 10, Figure 2) suggests control mechanisms in addition to those due to ArgR. Transcription of genes located close to the *cpk* cluster, as for the γ-butyrolactone-receptor (*scbR*) and the genes involved in γ-butyrolactone synthesis (*scbA, scbB*), was also affected by the Δ*argR* deletion and showed the same expression profile 10 (Figure 2; Table S5). Genes of the *act, red*, and *cpk* clusters are putatively under the control of ARG boxes (Table S4).

Secondary metabolism genes with expression altered in the Δ*argR* mutant include SCO7700 and SCO7701, which are involved in methylisoborneol biosynthesis, and *eshA* (SCO7699), a regulator of secondary metabolism (Saito et al., 2003, 2006); all of these show the expression profile of group 2. The *whiE* genes (SCO5314-5320), related to the synthesis of the TW95a pigment (Kelemen et al., 1998), are strongly upregulated in the Δ*argR* mutant (Table S5). A less pronounced effect (group 2, Figure 2) was observed in SCO1206 to SCO1208 genes for the synthesis of the tetrahydroxynaphtalene pigment (Table S5). The geosmine biosynthesis gene SCO6073 was underexpressed in the mutant at late times (49 to 66 h, Table S5). A secondary metabolite, ectoine, confers protection against osmotic stress and stabilizes proteins at high temperature and extreme pH to the cells (Bursy et al., 2008; Kol et al., 2010). The ectoine biosynthesis gene cluster (SCO1864 to SCO1867) was weakly overexpressed (1.5 to 3-fold) in the Δ*argR* mutant, showing the profile of group 3 (Table S5). A DNA fragment from the SCO1863-SCO1864 intergenic region was retarded *in vitro* by the ArgR protein (Table 1).

### Transcriptional analysis of genes involved in differentiation, sporulation, and gas vesicle formation

Clear expression differences were found in genes involved in hydrophobic cover formation (rodlins and chaplins), sporulation (*ram, whi*), cell wall glycan biosynthesis (*cwg*) and gas vesicle formation (*gvp*) (Table 2).

**Table 2:** Genes related to morphology differentially expressed in *S. coelicolor* M145 and *S. coelicolor* Δ*argR* (1).

**Code**	**Product**	**Gene**	**Mc** Δ***argR*****-M145**					***p*** **BH** Δ***argR*****-M145**				
			**32 h**	**42h**	**49h**	**56h**	**66h**	**32 h**	**42 h**	**49 h**	**56 h**	**66 h**
SCO0649	Putative gas vesicle synthesis protein	*gvpO2*	0.328	3.008	1.143	0.467	−0.183	0.856	**0.000**	0.021	0.557	0.870
SCO0650	Putative gas vesicle synthesis protein	*gvpA2*	0.274	3.122	1.713	0.522	1.138	0.868	**0.000**	**0.000**	0.414	0.011
SCO0651	Putative gas vesicle synthesis protein	*gvpF2*	0.230	1.843	0.510	0.216	0.035	0.799	**0.000**	0.092	0.681	0.967
SCO0652	Putative gas vesicle synthesis protein	*gvpG2*	0.632	2.872	1.248	0.227	0.106	0.381	**0.000**	**0.009**	0.803	0.929
SCO0653	Conserved hypothetical protein	*gvpY2*	0.023	1.157	−0.160	0.047	−0.012	0.999	**0.004**	0.715	0.964	0.992
SCO0654	Conserved hypothetical protein	*gvpZ2*	0.224	2.443	0.979	0.211	0.204	0.909	**0.000**	0.015	0.781	0.790
SCO0655	Putative gas vesicle synthesis protein.	*gvpJ2*	0.185	1.283	0.827	0.230	−0.231	0.945	**0.001**	0.030	0.754	0.736
SCO1415	Putative membrane protein	*smeA*	1.002	3.040	1.923	0.302	0.088	0.302	**0.000**	**0.006**	0.823	0.960
SCO1416	Putative membrane protein	*sffA*	0.738	1.982	1.474	0.449	0.310	0.066	**0.000**	**0.000**	0.353	0.517
SCO1489	BldD, transcriptional regulator of developmental genes	*bldD*	0.329	0.778	0.621	0.414	0.183	0.230	**0.000**	**0.004**	0.086	0.525
SCO1541	SsgA-like protein	*ssgB*	1.211	4.936	1.902	−0.010	−1.258	0.056	**0.000**	**0.001**	0.995	0.023
SCO1674	Putative secreted protein	*chpC*	0.522	1.634	0.363	0.484	−0.984	0.488	**0.000**	0.426	0.473	0.031
SCO1800	Putative small secreted protein	*chpE*	−0.016	0.082	−0.883	0.319	−0.490	0.999	0.848	**0.009**	0.520	0.182
SCO2082	Cell division protein	*ftsZ*	0.307	0.678	0.093	0.048	−0.159	0.244	**0.001**	0.658	0.915	0.578
SCO2716	Putative secreted protein	*chpA*	3.918	4.953	2.124	1.431	0.310	**0.000**	**0.000**	**0.000**	0.015	0.734
SCO2717	Putative small membrane protein	*chpD*	0.689	2.057	0.388	0.304	−1.100	0.609	**0.003**	0.592	0.822	0.141
SCO2718	Putative secreted protein	*rdlA*	5.182	6.836	3.209	2.305	0.705	**0.000**	**0.000**	**0.000**	**0.005**	0.486
SCO2719	Putative secreted protein	*rdlB*	4.621	5.711	2.595	1.667	0.496	**0.000**	**0.000**	**0.000**	0.018	0.596
SCO2786	beta-N-acetylhexosaminidase	*hexA*	0.345	0.305	−0.240	−0.283	−1.345	0.091	0.077	0.154	0.197	**0.000**
SCO3356	ECF sigma factor	*sigE*	0.042	−0.592	0.158	0.275	0.640	0.999	**0.001**	0.387	0.232	**0.001**
SCO3404	Cell division protein ftsH homolog	*ftsH2*	−0.007	0.548	0.059	0.067	0.249	0.999	**0.009**	0.807	0.883	0.330
SCO3925	IclR-type transcriptional regulator of ssgA	*ssgR*	0.594	1.519	0.329	−0.152	−0.429	0.159	**0.000**	0.322	0.815	0.271
SCO3926	Sporulation factor	*ssgA*	0.785	2.219	1.472	0.728	−0.671	0.127	**0.000**	**0.001**	0.164	0.139
SCO4035	RNA polymerase sigma factor	*sigF*	0.373	0.740	−0.049	−0.181	−0.407	0.394	**0.009**	0.888	0.717	0.206
SCO4767	Putative regulatory protein	*whiD*	−0.014	1.989	0.352	0.822	1.462	1.000	**0.001**	0.586	0.339	0.019
SCO4923	Conserved hypothetical protein		−0.203	−0.397	0.073	−0.171	0.038	0.232	**0.002**	0.584	0.330	0.896
SCO5046	Hypothetical protein	*wblI*	0.068	0.391	−0.258	−0.741	−1.258	0.999	0.126	0.302	**0.008**	**0.000**
SCO5240	Sporulation transcription factor-like	*wblE*	0.200	0.750	−0.189	−0.368	−0.704	0.706	**0.001**	0.422	0.222	**0.003**
SCO5314	whiE protein VII	*whiE-*ORFVII	0.333	5.188	2.766	0.383	0.223	0.942	**0.000**	**0.000**	0.768	0.890
SCO5315	polyketide cyclase	*whiE-*ORFVI	0.469	2.869	1.951	0.814	0.034	0.741	**0.000**	**0.001**	0.306	0.983
SCO5316	acyl carrier protein	*whiE-*ORFV	1.023	5.555	2.857	0.694	0.454	0.270	**0.000**	**0.000**	0.500	0.679
SCO5317	polyketide beta-ketoacyl synthase beta	*whiE-*ORFIV	0.023	1.395	0.409	−0.087	−0.314	0.999	**0.003**	0.388	0.941	0.683
SCO5318	polyketide beta-ketoacyl synthase alpha	*whiE-*ORFIII	0.143	3.152	1.263	−0.178	0.024	0.995	**0.000**	**0.008**	0.851	0.985
SCO5319	whiE protein II	*whiE-*ORFII	0.245	2.398	1.389	0.101	0.216	0.960	**0.000**	0.014	0.944	0.860
SCO5320	whiE protein I	*whiE-*ORFI	0.083	2.182	0.777	0.100	0.030	0.999	**0.000**	0.073	0.928	0.981
SCO5321	polyketide hydroxylase	*whiE-*ORFVIII	0.023	1.479	0.325	−0.024	−0.125	0.999	**0.000**	0.374	0.980	0.877
SCO5580	Putative prokaryotic docking protein	*ftsY*	0.058	0.027	0.290	0.242	0.746	0.999	0.938	0.233	0.526	**0.003**
SCO5819	Sporulation transcription factor, WhiH	*whiH*	1.978	3.388	1.448	1.197	−0.647	**0.000**	**0.000**	**0.004**	0.026	0.241
SCO6029	Two-component regulator	*whiI*	2.696	3.526	1.840	1.287	0.261	**0.000**	**0.000**	**0.002**	0.039	0.807
SCO6131	Putative carboxypeptidase		0.039	−0.815	−0.648	0.002	0.563	0.999	**0.001**	0.010	0.998	0.027
SCO6180	Putative transferase	*cwgB*	0.225	1.275	1.786	1.779	1.527	0.572	**0.000**	**0.000**	**0.000**	**0.000**
SCO6181	Putative transferase	*cwgC*	0.047	0.777	1.267	1.303	1.058	0.999	**0.001**	**0.000**	**0.000**	**0.000**
SCO6182	Putative dehydratase	*cwgD*	0.148	0.396	0.682	1.108	0.695	0.822	0.061	**0.002**	**0.000**	**0.002**
SCO6183	Putative transferase	*cwgE*	0.325	0.084	1.086	1.551	1.017	0.492	0.817	**0.000**	**0.000**	**0.001**
SCO6185	Putative transferase	*cwgG*	0.361	1.704	2.092	1.867	1.200	0.341	**0.000**	**0.000**	**0.000**	**0.000**
SCO6186	Putative phosphoheptose isomerase	*cwgH*	0.592	0.846	1.200	0.942	0.746	0.148	**0.009**	**0.001**	**0.010**	0.025
SCO6187	Putative bifunctional synthase/transferase	*cwgI*	0.313	0.338	0.943	0.896	0.213	0.573	0.286	**0.003**	**0.007**	0.652
SCO6188	Putative transferase	*cwgJ*	0.087	0.440	0.826	0.766	0.627	0.990	0.111	**0.004**	0.011	0.024
SCO6499	Putative gas vesicle synthesis protein	*gvpO*	0.185	0.799	1.259	1.507	2.420	0.990	0.170	0.023	0.018	**0.000**
SCO6500	Putative gas vesicle synthesis protein	*gvpA*	0.163	0.669	1.206	1.464	2.244	0.973	0.108	**0.004**	**0.001**	**0.000**
SCO6501	Putative gas vesicle synthesis protein	*gvpF*	0.196	0.720	0.843	1.014	1.662	0.836	0.011	**0.005**	**0.001**	**0.000**
SCO6502	Putative gas vesicle synthesis protein	*gvpG*	0.263	0.787	0.602	0.923	1.691	0.879	0.068	0.152	0.065	**0.000**
SCO6503	Hypothetical protein SC1E6.12	*gvpY*	0.126	0.396	0.521	0.767	0.966	0.978	0.271	0.114	0.049	**0.006**
SCO6504	Conserved hypothetical protein SC1E6.13	*gvpZ*	0.323	0.781	0.777	1.201	1.910	0.722	0.039	0.035	**0.005**	**0.000**
SCO6505	Putative gas vesicle synthesis protein	*gvpJ*	−0.077	0.408	0.768	1.036	1.300	0.999	0.183	0.011	**0.002**	**0.000**
SCO6506	Putative gas vesicle protein	*gvpL*	0.172	−0.088	0.420	0.645	1.143	0.860	0.796	0.104	0.033	**0.000**
SCO6507	Putative gas vesicle synthesis protein	*gvpS*	−0.375	−0.076	−0.440	0.416	0.955	0.391	0.840	0.109	0.284	**0.001**
SCO6682	Hypothetical protein SC5A7.32	*ramS*	−2.158	−0.491	−0.152	−1.645	−1.481	**0.004**	0.532	0.854	0.034	0.036
SCO6685	Two-component system response regulator	*ramR*	−0.570	−0.394	−1.116	−0.741	−0.697	0.091	0.177	**0.000**	0.019	0.016
SCO6715	Putative transcriptional regulator	*wblH*	−0.505	0.203	−0.231	0.671	2.427	0.817	0.828	0.775	0.549	**0.001**
SCO7050	Putative D–alanyl-D-alanine carboxypeptidase		0.437	0.851	0.460	0.249	0.169	0.306	**0.004**	0.111	0.605	0.750
SCO7257	Putative secreted protein	*chpB*	1.118	2.174	0.504	0.415	−0.223	0.017	**0.000**	0.221	0.520	0.772
SCO7699	EshA protein	*eshA*	1.043	2.524	0.185	−0.018	−2.170	0.030	**0.000**	0.688	0.988	**0.000**

*(1) For each gene, the Mc value is the binary log of the differential transcription between the mutant and the wild strain. A positive Mc value indicates upregulation, and a negative one, downregulation. Data are the average of three biological replicates and Benjamini-Hochberg adjusted p-values are indicated. Bold numbers indicate p-values below the threshold (0.01) set to identify significantly diferentially expressed genes*.

#### Genes for rodlins and chaplins

**Figure 5:**
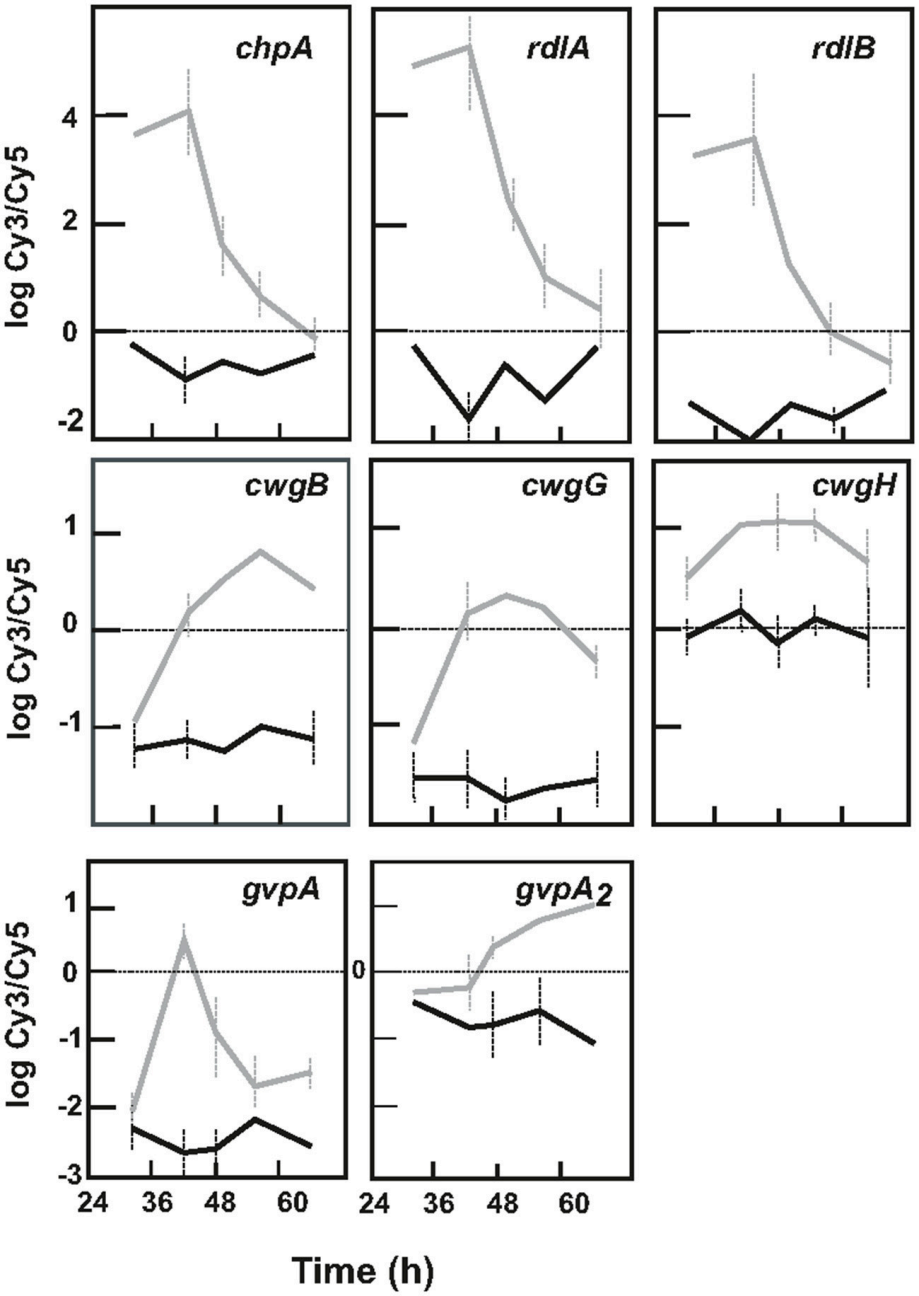
Expression of genes related to differentiation in *S. coelicolor* M145 and *S. coelicolor* Δ*argR*. **(Upper panels)** Expression profile of genes encoding chaplins (*chpA* as model) and rodlins (*rdlA, rdlB*). **(Middle panels)** Expression profile of *cwg* genes. Expression of *cwgB, cwgG*, and *cwgH* are shown as model. **(Lower panels)** Expression of genes for gas vesicles: *gvpA* and *gvpA*_2_ are shown as model of genes for gas vesicle clusters I and II. *S. coelicolor* M145 genes (black lines), *S. coelicolor* Δ*argR* genes (gray lines). Standard deviation is represented by discontinuous bars.

The rodlet layer formed by chaplins and rodlins (Claessen et al., 2004) is partially responsible for the hydrophobicity in aerial hyphae and spores. The genes encoding chaplins (*chpA, chpB, chpC, chpD*, and *chpG*) and rodlins (*rdlA* and *rdlB*) were upregulated in the Δ*argR* mutant. This overexpression was particularly high in the exponential growth phase (Table 2). The *chpA, rdlB*, and *rdlA* gene expression increased 30-, 52-, and 114-fold, respectively, at 42 h (Figure 5, upper panels), while *chpC* and *chpB* were less affected (4.5-fold increase, Table 2).

#### Cwg genes

The *cwg* cluster (SCO6179 to SCO6190) is tentatively involved in glycan cell wall synthesis (Hong et al., 2002). All *cwg* genes were overexpressed (1.3 to 4.3-fold) in the Δ*argR* mutant (Figure 5, middle panels).

#### Genes for gas vesicles

Two independent gene clusters involved in gas vesicle formation (*gvp* genes) were present in the *S. coelicolor* M145 genome. Both clusters showed low and relatively constant expression along the developmental time course in the control strain. However, in cultures of *S. coelicolor* Δ*argR*, all *gvp* genes were overexpressed. Cluster I gene expression (SCO0649-SCO0657) slowly increased and peaked at 42 h, as shown for the model gene *gvpA* (Figure 5, lower left panel). Transcription for genes in cluster II (SCO6499-SCO6508) increased along with time in the mutant, showing 2- to 4-fold higher expression than in the control strain in the stationary phase; as a prototype gene of cluster II, *gvpA*_2_ expression is shown in Figure 5 (right lower panel).

#### Genes related to spore formation and differentiation

**Figure 6:**
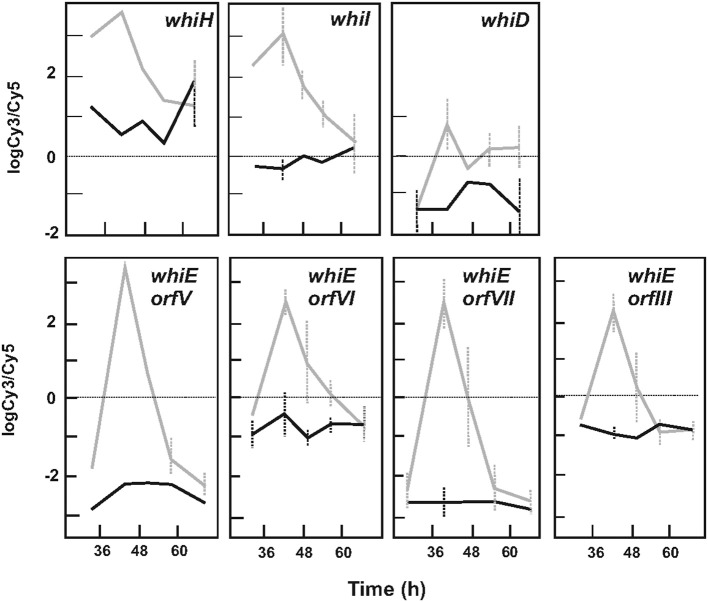
Expression profile of *whi* genes in *S. coelicolor* M145 and *S. coelicolor* Δ*argR*. **(Upper panels)** Expression of *whiH, whiI*, and *whiD* genes. **(Lower panels)** Expression of *whiE* cluster genes. The *whiE-orfV, orfVI, orfVII*, and *orfIII* are shown as models. *S. coelicolor* M145 genes (black lines), *S. coelicolor* Δ*argR* genes (gray lines). Standard deviation is represented by discontinuous bars.

All *whi* genes were involved in sporulation and aerial hyphae formation (Davis and Chater, 1992), with the exception of *whiJ* and *whiA*, which were significantly upregulated by the absence of ArgR (Table 2). The *whiD, whiI*, and *whiH* genes (Figure 6, upper panels) and the eight *whiE* genes (*orfI* to *orfVIII*), which are involved in the formation of the spore pigment (Kelemen et al., 1998) (Figure 6, lower panels), were most overexpressed in the mutant. The ARG box located in the *whiB* promoter region (R*i* 9.4 bits, Table 1) was demonstrated to bind ArgR in previous DNA band-shift studies (Pérez-Redondo et al., 2012), and several *whi* genes are under the predicted control of ARG boxes (Table S4).

The *ramR* gene, which encodes an orphan response regulator related to the SapB peptide and aerial mycelium formation (San Paolo et al., 2006), was weakly down-regulated in the Δ*argR* mutant (Table S5). The transcription of genes for key sporulation regulatory proteins, such as *ssgR, ssgA, ssgB*, and *smeA-sffA* (van Wezel et al., 2000a; Traag et al., 2004; Ausmees et al., 2007; Willemse et al., 2011), were all upregulated in the mutant (Table 2).

Other genes related to morphological differentiation had smaller, but significant, differences in expression between the parental and Δ*argR* mutant strains. These were the developmental transcriptional regulator *bldD* (Elliot et al., 2001), which presents functional ARG boxes upstream of its coding sequence (Pérez-Redondo et al., 2012); *wblH*, a target of WhiA (Bush et al., 2013); and *ftsZ*, a key protein of cell division (van Wezel et al., 2000b; Bush et al., 2013). All weakly increased w their expression at the first three sampling times (Table S5). Also upregulated in the Δ*argR* mutant were *hexA*, encoding for an N-acetylhexosaminidase involved in glycan degradation (Mark et al., 1998); SCO4923, putatively involved in septum formation; SCO7050, for a D,D-carboxypeptidase-like protein; and SCO7699, reported to be involved in sporulation (Table 2).

### Analysis of *S. coelicolor* M145 and Δ*argR* mutant differentiation

**Figure 7:**
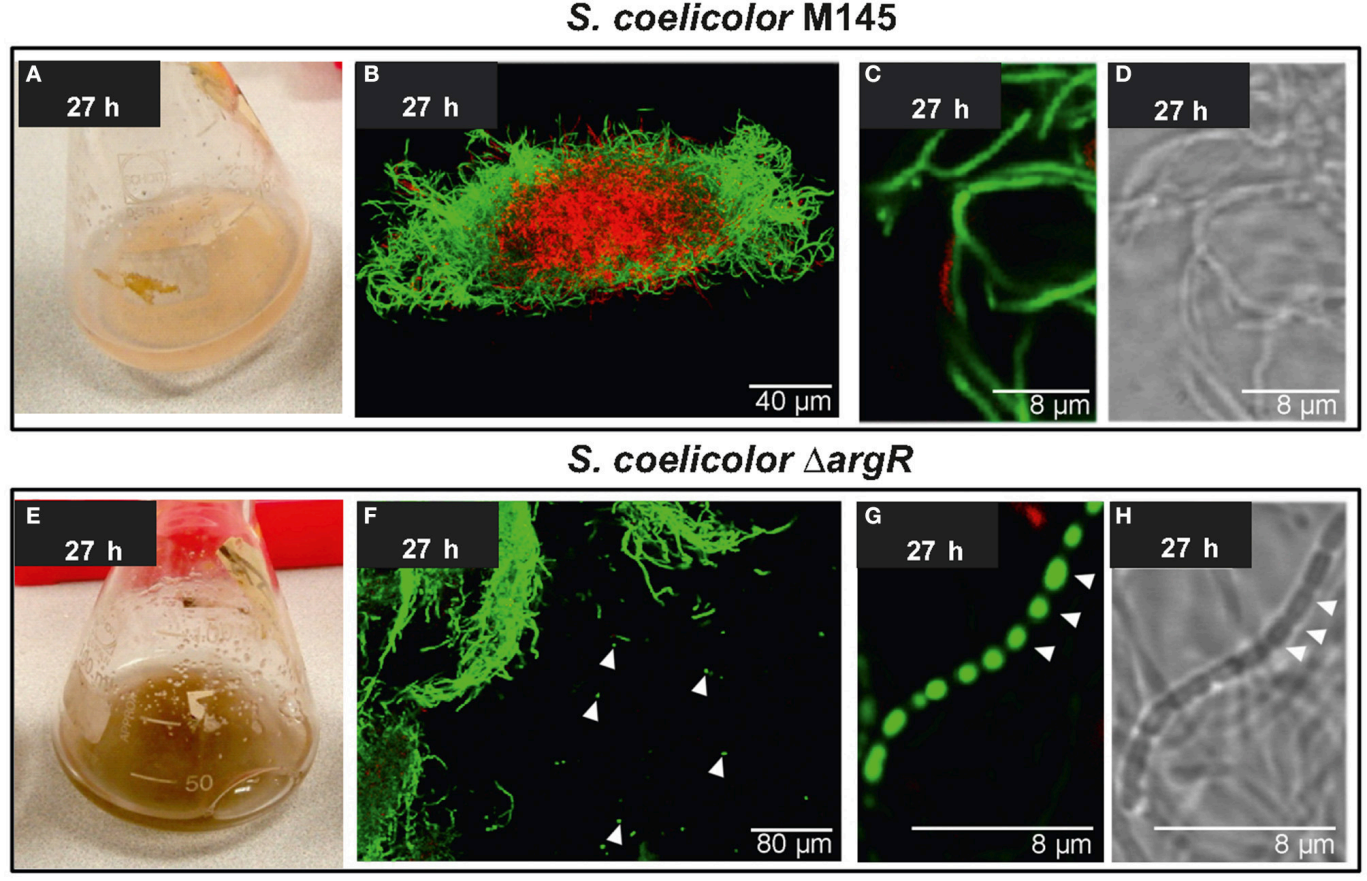
Analysis of differentiation of *S. coelicolor* M145 and *S. coelicolor* Δ*argR. Streptomyces coelicolor* M145 (upper panels) and *Streptomyces coelicolor* Δ*argR* (lower panels). **(A,E)** Macroscopic differences in color between control and mutant strain. **(B–D, F–H)** Confocal laser-scanning fluorescence microscopy analysis (SYTO9/PI staining) of strains growing in liquid MG medium. **(D,H)** correspond to interference contrast mode images. Arrowheads indicate spore-like structures.

The mycelium from liquid cultures of the Δ*argR* mutant showed a dark, brownish pigment, which was not observed in the *S. coelicolor* M145 mycelium (compare Figure 7A with Figure 7E). Morphological differentiation was analyzed in liquid cultures using confocal microscopy. The most important difference between *S. coelicolor* M145 and the Δ*argR* mutant was the presence of nucleoid segregation (Figure 7G), and the formation of round segments (Figure 7H) with an average length of 0.9 μm ± 0.1 in the Δ*argR* mutant, resembling the segmentation observed during sporulation in solid cultures. Hypha segmentation began at 27 h of culture and affected 4.3% ± 0.1 of the hyphae (Figure S1).

### Validation of transcriptomic data by qRT-PCR

**Figure 8:**
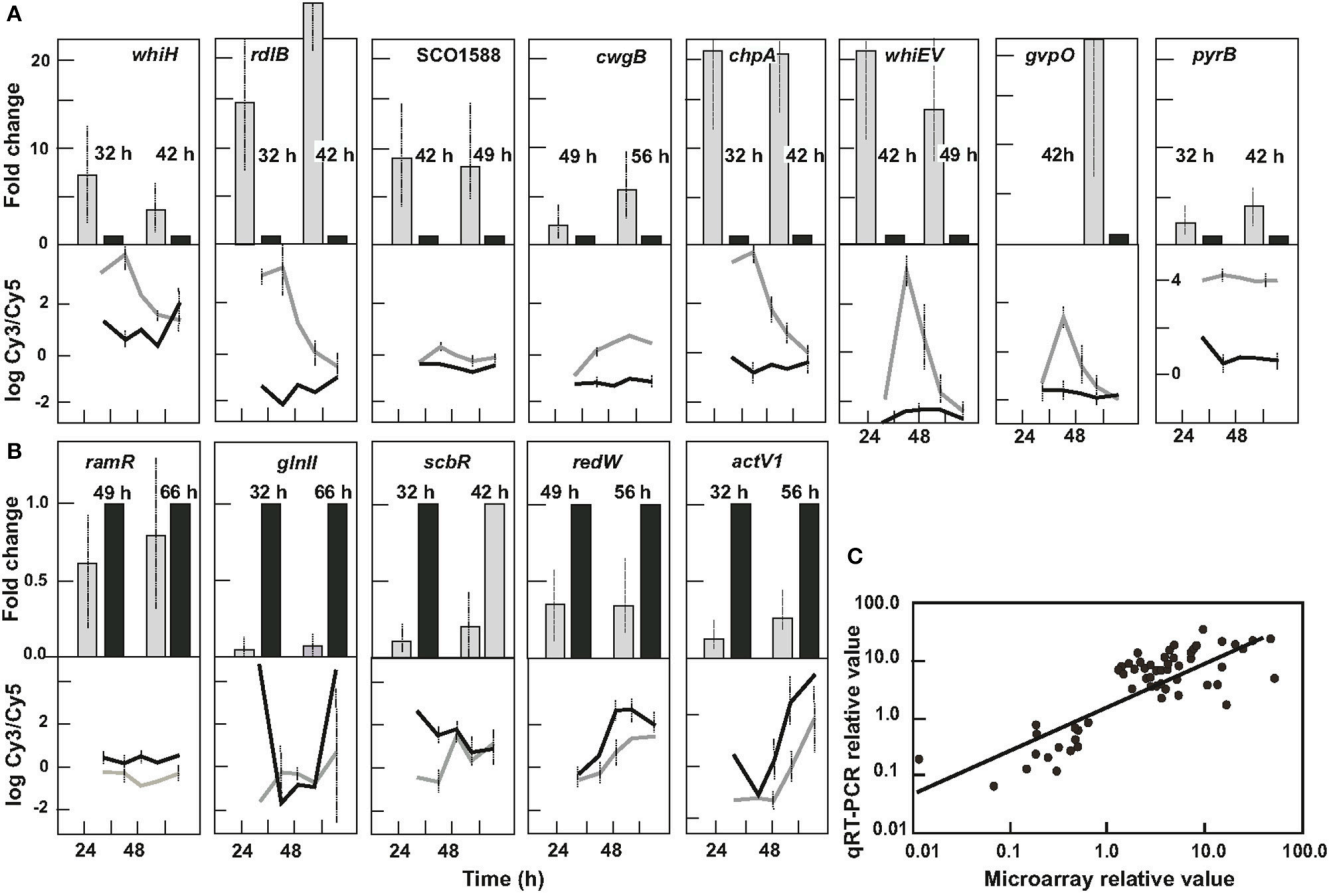
Validation by qRT-PCR of microarray data, corresponding to genes involved in primary metabolism, secondary metabolism, and differentiation. **(A)** Validation of genes overexpressed in Δ*argR* mutant. **(B)** Validation of genes underexpressed in Δ*argR* mutant. The expression is presented in relation to the control strain, taken as 1. Upper panel indicate gene name and culture time at which qRT-PCR was performed. Corresponding lower panel shows gene profile of the microarray study. Black bars and black lines correspond to control strain *S. coelicolor* M145. Gray bars and gray lanes correspond to *S. coelicolor* Δ*argR*. **(C)** qPCR vs. microarray: Scatter plot correlating to transcriptomic changes as measured by microarray analysis and qRT-PCR of all genes validated. *R*^2^ was 0.926.

Transcriptomic data were validated using qRT-PCR at two developmental time points for thirteen of the genes related to differentiation and secondary metabolism: *whiH, rdlB*, SCO1588, *cwgB, chpA, whiE-orfV, gypO, pyrB, ramR, glnII, scbR, redW*, and *actV1* (Figure 8). The expression levels of 14 additional genes from all the expression profiles shown in Figure 1 were also validated (Figure S2). The correlation between the qRT-PCR and microarray results for the 27 genes was very good, with an *R*^2^ value of 0.926 (Figure 8), confirming the reliability of the transcriptomic data.

## Discussion

The differences in the transcriptomes of *S. coelicolor* M145 and the Δ*argR* mutant strain were previously studied at a single developmental time point (Pérez-Redondo et al., 2012). The current studies were extended by analyzing five different developmental stages in the culture.

The existence of ARG boxes in ArgR controlled genes suggests a modulator role of ArgR in the transcription. The information content (R*i*) of the operators formed by a single ARG box, listed in Table 1, ranged from 1.2 bit (*afsR*) to 14.5 bit (SCO4293). The presence of one or two ARG boxes, the distance between them, the R*i* value, and their location in relation to other regulatory signals, may account for different ArgR binding affinities and allow fine-tuned regulation of the expression of the controlled genes. ARG boxes were predicted in the genome using the new model (Table S4). Those sites, if functional, might account for the altered transcription in the Δ*argR* mutant. Alternatively, in the absence of an ArgR binding site, the regulatory role of ArgR in the expression would be indirect.

This study demonstrates that ArgR is a pleiotropic regulator, which in *S. coelicolor* represses more than the genes for arginine and pyrimidine biosynthesis (Cunin et al., 1983; Larsen et al., 2008). A total of 1,544 genes out of the 7,721 analyzed were significantly deregulated at least once according to the microarray experiment (Table S5). Forty-five genes were always overexpressed (e.g., at 5 time points) in the ArgR mutant (Table S5), which suggests that the ArgR protein exerts a tight control over their transcription. Most of them, 29 out of these 45 genes (64%) had the profile of group 1 (Figure 2), including the 15 genes related to arginine and pyrimidine biosynthesis. The other 16 genes did not fit any of the 10 groups determined by maSigPro. The function of many of these 29 genes is unknown, although SCO6824-SCO6827 resembles a polyketide synthesis gene cluster and *sigM* (SCO7314) has been reported to be involved in osmotic stress control (Lee et al., 2005). ArgR direct control over some of these 45 deregulated genes was demonstrated by binding to functional ARG boxes located upstream of SCO1086, *pyrA, pyrB, pyrR, bldD, argH, argC, arcB, argG*, and *sigM* (Table 1), and non-tested, but predicted ARG boxes could account for the control of SCO2864-SCO2869, SCO6205-SCO6206, SCO6824-SCO6827 genes (Table S4). However, most of the deregulated genes (1499) were over- or under-expressed at one, two, three or four time points, indicating a ArgR relaxed control and/or interaction with other regulators. The nitrogen metabolism genes are controlled in *S. coelicolor* by GlnR, the global regulator of nitrogen assimilation, by NnaR (Amin et al., 2012) and also by PhoP, the global regulator of phosphate metabolism (Rodríguez-García et al., 2009; Sola-Landa et al., 2013). We found that, in addition, some nitrogen metabolism genes (*glnII, amtB, glnK, glnD*) are regulated by ArgR. This was a direct effect, since expression of *glnR* and *phoP* was not affected in the Δ*argR* mutant. Arginine is a nitrogen-rich storage compound in many organisms (Llácer et al., 2008), and the discovery of ArgR binding-ARG boxes (Pérez-Redondo et al., 2012) in the *glnR* and *amtB* genes, supports a direct regulatory role for ArgR in nitrogen metabolism.

A similar situation occurs in cell wall biosynthesis genes (*cwg*), which were upregulated in the Δ*argR* mutant. The *cwg* genes were predicted to be transcribed from the *cwgA* upstream promoter, which is controlled by SigE (Hong et al., 2002) in response to the signal transmitted by the two component system CseC-CseB (Paget et al., 1999). Our results suggest an additional ArgR regulation of *cwg* genes. Expression of genes for secondary metabolite biosynthesis was also altered in the Δ*argR* mutant. The *act* and *red* genes were repressed, and genes for the TW95a pigment, and coelymicin were overexpressed. The genes for ectoine biosynthesis, controlled by GlnR (Shao et al., 2015), were overexpressed at all time points.

In *S. coelicolor* the *gvp* genes for a putative regulator and gas vesicle structural proteins are located in two duplicated clusters. These gas-filled vesicles are required in aquatic organisms for flotation, but have never been found in soil-dwelling bacteria and the presence of these gene clusters (Offner et al., 1998) is surprising. In fact, disruption of the *gvp* gene clusters does not affect the buoyancy of *Streptomyces* cells in liquid cultures (van Keulen et al., 2005). An induction of *gvp* genes was found following exposure to high concentrations of salt, and it has been proposed that gas vesicles may counteract hyperosmotic stress. The expression of both *S. coelicolor gvp* clusters was upregulated in the Δ*argR* strain. The Gvp proteins have a very high content of arginine, glutamate, and proline, up to 42% of the protein total amino acids in GvpA_2_, and might have evolved in soil *Actinobacteria* as nitrogen storage material, which might explain the ArgR control on their biosynthesis.

Some putative ArgR binding sequences were found in these differentially expressed genes, including ARG boxes located in coding regions. The presence of binding sequences for regulatory proteins in coding sequences is not unusual; in a study of the PHO regulon in *S. coelicolor*, almost 70% of the PhoP-chromatin-immunoprecipitated enriched fragments, and ~50% of the bioinformatically located PHO boxes, were located in coding sequences (Allenby et al., 2012).

DNA band-shift studies using ArgR protein identified 24 regions containing ARG boxes (Pérez-Redondo et al., 2012). Here, we show additional DNA fragments bound by ArgR *in vitro* (Figure 1A). However, other DNA fragments tested with putative ARG boxes showed no band retardation on EMSA (Table S3). The lack of binding in sequences putatively involved in regulation is not rare. The presence of ARG boxes might not be sufficient indicator of affinity binding *in vitro*. In fact, Sola-Landa et al. (2008) selected, with very stringent criteria, 20 promoters containing PHO boxes but were able to confirm the functionality of only 40% of them using an *in vitro* gel-shift assay. A lack of binding may derive from low Ri boxes but also depends on the correct spatial configuration of the DNA in the fragment used, the *in vivo* requirement of additional accessory proteins or cofactors that increase the affinity binding of the regulatory protein, and/or the requirement of *in vivo* modifications of the binding regulator (Wade et al., 2007). This is the case for RocR and AhrC cooperation in *Bacillus* to activate expression of arginine catabolism genes (Gardan et al., 1997) or the cooperation of the regulatory proteins FarR and ArgR in corynebacteria, to control *argB* expression and intracellular ornithine levels (Lee et al., 2011).

The relationship between ArgR and hyphae differentiation remained unexplored. As detailed above, *S. coelicolor* Δ*argR* mutants showed a spectacular phenotype in liquid cultures, resulting in the formation of spore-like chains. Microscopy analysis displayed the division and separation of nucleoids and the physical strangulation of hypha, forming chains of individual round segments in mutant liquid cultures, two principal events associated with sporulation (Figure 7). While sporulation in liquid cultures has occasionally been described in other *Streptomyces* strains (Lee and Rho, 1993; Rho and Lee, 1994; Rueda et al., 2001), in *S. coelicolor* it is very unusual and has only been reported in flask cultures submitted to either nutritional downshift or Ca^2+^supplementation (Daza et al., 1989), in *S. coelicolor* strains overexpressing *ssgA* (van Wezel et al., 2000a), and recently, in 2-L bioreactors (Rioseras et al., 2014). The signals triggering sporulation in *Streptomyces* hyphae (the upper parts of the aerial mycelia in solid cultures; the border of the mycelial pellets in liquid cultures) remain poorly characterized. Our results suggest that the arginine metabolism can contribute to modulate sporulation.

The differentiation signals activating sporulation and secondary metabolism are not completely known, especially in liquid cultures (Salerno et al., 2013). This work suggests that ArgR contributes to the regulation of these processes blocking sporulation in liquid cultures of the parental strain. In addition, this phenotype correlates with the overexpression in the Δ*argR* mutant of genes involved in hydrophobic cover formation, differentiation, and sporulation (e.g., chaplins, rodlins, most *whi* genes, *ramR, ssgR, ssgA, smeA-sffA*). Several possible ARG boxes were associated with the *whi* genes, suggesting a direct interaction with ArgR. In the case of rodlin and chaplin genes, no putative regulatory sequences were bioinformatically detected, indicating possible indirect regulation through other genes. Small but significant differences were found for some genes related to sporulation, such as *sigF* (Kelemen et al., 1996), or to hyphae septation, such as *ftsZ* and *ftsH2*, but not for other genes involved in the formation of the cytokinetic Z ring (*ftsW, ftsI, ftsQ*), (Grantcharova et al., 2003). No significant differences were found for other genes, such as rodlin and chaplin genes, genes related to aerial mycelium formation (*ramCSAB*) (Keijser et al., 2002; O'Connor and Nodwell, 2005), hyphae elongation or cellular division (*whiA, crgA*) (Flärdh et al., 1999). The regulatory genes differentially expressed in the Δ*argR* mutant with respect to the *S. coelicolor* parental strain are potential regulators of sporulation-like processes detected in liquid cultures. Further work is necessary to achieve a deeper characterization of the biochemical mechanism behind activation of sporulation in liquid cultures.

In summary, this work demonstrates that the ArgR protein is more pleiotropic than other bacterial ArgRs, affecting the expression of 1544 genes and triggering a sporulation-like process under the growth conditions used in this work. A new weight matrix was developed for the identification of novel ARG boxes, and a database containing the expression data of genes differentially expressed in the Δ*argR* mutant was generated.

## Author contributions

PL, AR-G, and AM: conceived and designed research. AB, RP-R, RÁ-Á, and PY: performed research. AR-G: did the bioinformatics studies. PY and AM: did the differentiation studies. PL: wrote the manuscript.

### Conflict of interest statement

The authors declare that the research was conducted in the absence of any commercial or financial relationships that could be construed as a potential conflict of interest.
