# Supplementary material for: ArgR of Streptomyces coelicolor Is a Pleiotropic Transcriptional Regulator: Effect on the Transcriptome, Antibiotic Production, and Differentiation in Liquid Cultures
Source: Front Microbiol. 2018 Mar 1;9:361. doi: 10.3389/fmicb.2018.00361 (PMC5839063; doi:10.3389/fmicb.2018.00361)
Supplement: Supplementary file 1 [file Table1.PDF]

Table S1. Estimation of spot weights for microarray data analysis

The Feature Extraction software that was used to quantify the spot fluorescence also provided a set of Boolean values to assess the data quality of each spot for both the red and green channels. A combination of these quality values were summarized into a quality flag (A-F) as indicated in the table below. Please note that 99.3 % of the spots were of the highest quality. To obtain spot quality weights (0-1 values that can be entered into the data analysis), we followed the procedure described previously [Rodríguez-García et al., 2007]. The results are summarized in the table bellow. These weights were applied for the set of 35 621 valid probes. The rest of probes were assigned an extremely low weight (10-6) to discard their influence in the normalization and linear model processes.

Rodríguez-García, A., Barreiro, C., Santos-Beneit, F., Sola-Landa, A. and Martín, J.F. (2007). Genome-wide transcriptomic and proteomic analysis of the primary response to phosphate limitation in *Streptomyces coelicolor* M145 and in a Δ*phoP* mutant. *Proteomics* 7, 2410–2429.

gIsPosAndSignif	rIsPosAndSignif	gIsFeatNonUnifOL	rIsFeatNonUnifOL	gIsBGNonUnifOL	rIsBGNonUnifOL	**Quality flag**	**Total number of spots (30 microarrays)**	**Quality weight**
**1**	**1**	**0**	**0**	**0**	**0**	**A**	1304616	1.0000000
**1**	**1**	**1**				**B**	7675	0.7708824
**1**	**1**	**>=2**				**C**	370	0.3723605
**1**	**0**					**D**	0	
**0**	**1**					**E**	1233	0.4711404
**0**	**0**					**F**	46	0.0833845
