# Supplementary material for: ArgR of Streptomyces coelicolor Is a Pleiotropic Transcriptional Regulator: Effect on the Transcriptome, Antibiotic Production, and Differentiation in Liquid Cultures
Source: Front Microbiol. 2018 Mar 1;9:361. doi: 10.3389/fmicb.2018.00361 (PMC5839063; doi:10.3389/fmicb.2018.00361)
Supplement: Supplementary file 2 [file Table2.PDF]

Table S2. Oligonucleotides used in this work [1, 2]

Primer	Sequence (5' - 3')	Use
**CAR140**	ACACCGAGGATGTGGCCG	FP for SCO0464 qRT-PCR analysis
**CAR141**	CGAACTGGAGGCGCTCGT	RP for SCO0464 qRT-PCR analysis
**CAR142**	GGTGCGGTGGACCAGGGA	FP for *glpK2* qRT-PCR analysis
**CAR143**	GCGCGGGAGGATCTGCTC	RP for *glpK2* qRT-PCR analysis
**CAR144**	ACGGCCCGTACGAGATCC	FP for SCO0524 qRT-PCR analysis
**CAR145**	CCAGACCAGCTGACGCCA	RP for SCO0524 qRT-PCR analysis
**CAR146**	CGCCACGTCGGTAGAGGA	FP for SCO0870 qRT-PCR analysis
**CAR147**	GAGCTGATGACCCGCGAG	RP for SCO0870 qRT-PCR analysis
**CAR148**	GCCGGGCGAGGTGCTCGT	FP for *inhA* qRT-PCR analysis
**CAR149**	CCGCGAAGCTGGCCCAGG	RP for *inhA* qRT-PCR analysis
**CAR150**	TTCGTGGCGGCCCCGTCC	FP for *chpA* qRT-PCR analysis
**CAR151**	CGCCGCGACGGGCATCCT	RP for *chpA* qRT-PCR analysis
**CAR152**	GGACCAGGGCCACCCGCG	FP for *actVA1* qRT-PCR analysis
**CAR153**	GCGCCCAGGCCCTGCTCG	RP for *actVA1* qRT-PCR analysis
**CAR154**	GTCCAGGCCGAAGGTGTC	FP for *whiE* ORFV qRT-PCR analysis
**CAR155**	GGTGACCGTCGAGGAGCT	RP for *whiE* ORFV qRT-PCR analysis
**CAR156**	CGAACGGCGGCTGATCTG	FP for SCO5658 qRT-PCR analysis
**CAR157**	TCGAAGGCCTCCAGCGTG	RP for SCO5658 qRT-PCR analysis
**CAR158**	GCCGAGGACGGAGGTGCC	FP for *recX* qRT-PCR analysis
**CAR159**	GGTCCCGGTGAGCAGGCG	RP for *recX* qRT-PCR analysis
**CAR160**	CCGGCGCGAGCTGGACTC	FP for *redW* qRT-PCR analysis
**CAR161**	GTCCAGTCCGCTGCCGCC	RP for *redW* qRT-PCR analysis
**CAR164**	GCGGCGATCAACCCCTTC	FP for *gcl* qRT-PCR analysis
**CAR165**	TAGCCCTCGGCCATGTGC	RP for *gcl* qRT-PCR analysis
**CAR170**	ACCTCGGACAGCAGCTGC	FP for *tktA2* qRT-PCR analysis
**CAR171**	GGTTGGCCAGCAGTACGG	RP for *tktA2* qRT-PCR analysis
**CAR172**	AGGTGCGGATCCTCAAGG	FP for *gvpF* qRT-PCR analysis
**CAR173**	TCAGCACGTTCTGGTGGG	RP for *gvpF* qRT-PCR analysis
**CAR174**	TACCACCCAGTCCGCCCA	FP for *sigM* qRT-PCR analysis
**CAR175**	CGTGACGAGTTCGTCGCG	RP for *sigM* qRT-PCR analysis
**CAR176**	AAGGTGGTCCGGACGGTG	FP for *pepA* qRT-PCR analysis
**CAR177**	GAAGAGCGAAGTCGCCCG	RP for *pepA* qRT-PCR analysis
**CAR180**	GTGCGTCACCTCGAAGTG	FP for *katA2* qRT-PCR analysis
**CAR181**	ACGCCTACCTCATCGAGC	RP for *katA2* qRT-PCR analysis
**CAR182**	GCCCGACCTCAGCGTCTT	FP for *ectB* qRT-PCR analysis
**CAR183**	GTACATGCGGCTGCCGAC	RP for *ectB* qRT-PCR analysis
**CAR184**	TGCTGGAGAGGCCGACGG	FP for *lexA* qRT-PCR analysis
**CAR185**	CAGCGCCGGGTGATCGAG	RP for *lexA* qRT-PCR analysis
**CAR186**	GTACGCTCCCGCCGGAGA	FP for *hrdB* qRT-PCR analysis
**CAR187**	GAAGGCCCGACGCACGTC	RP for *hrdB* qRT-PCR analysis

**CRP81**	TCCTCCTCTTCCGCCGGGAATC	FP to obtain the *afsR* probe for EMSA
**CRP82**	CCTCCCCCGCCTCAACAGCGT	RP to obtain the *afsR* probe for EMSA
**CRP83**	TGTGGCGTCCGCGTCCTTC	FP to obtain the *afsS* probe for EMSA
**CRP84**	CCGGAGCGTTCAGCGTTC	RP to obtain the *afsS* probe for EMSA
**ramR_F**	ACTCTCCGTCCACGACACAG	FP for *ramR* qRT-PCR analysis
**ramR_R**	GCACTGGTGCAACTGCTC	RP for *ramR* qRT-PCR analysis
**argC_F**	GGCCAGCGGCAGCAGATGC	FP for *argC* qRT-PCR analysis
**argC_R**	GGTGGCCGGAGCGAGTGG	RP for *argC* qRT-PCR analysis
**pyrB_F**	GTGAAGTTGATGACGTCCG	FP for *pyrB* qRT-PCR analysis
**pyrB_R**	CGATCAAGAAACTGCCGA	RP for *pyrB* qRT-PCR analysis
**glnII_F**	CCAAGCTCCGTTCCAAGA	FP for *glnII* qRT-PCR analysis
**glnII_R**	AGAAGACCGGCTTGAGCA	RP for *glnII* qRT-PCR analysis
**rdlB_F**	GACTGGCCAGTGATCAAGAA	FP for *rdlB* qRT-PCR analysis
**rdlB_R**	ATGTTGCCCGTGGTCATC	RP for *rdlB* qRT-PCR analysis
**gvpO_F**	ACCTCGAAGGAGTCTCCG	FP for *gvpO* qRT-PCR analysis
**gvpO_R**	AGCAGACTGGTGGTGTCG	RP for *gvpO* qRT-PCR analysis
**cwgB_F**	CGAGGAAGAGCGGTTCAG	FP for *cwgB* qRT-PCR analysis
**cwgB_R**	TCGAAGGAGTAGGCGACG	RP for *cwgB* qRT-PCR analysis
**whiH_F**	AGATCGGACAGCGTTTCG	FP for *whiH* qRT-PCR analysis
**whiH_R**	TCCAGCAGGTTCCAGTCA	RP for *whiH* qRT-PCR analysis
**scbR_F**	GCAAGCGGTGACAGAACA	FP for *scbR* qRT-PCR analysis
**scbR_R**	CGATCACGGAGATCCTCA	RP for *scbR* qRT-PCR analysis
**redH_F**	GCGACCTCGCCTGGTTCC	FP to obtain the *redH* probe for EMSA
**redH_R**	TCGTTCTTGGCGGCCTGG	RP to obtain the *redH* probe for EMSA
**SCO5326_F**	GCGTAGGTGACGCTCCATCA	FP to obtain the SCO5326 probe for EMSA
**SCO5326_R**	AGCATGGTGGCCCACTCC	RP to obtain the SCO5326 probe for EMSA
**eshA_F**	CGAAGCCCAGCCGTCGATAG	FP to obtain the *eshA* probe for EMSA
**eshA_R**	TAGTCGGGGCCGTGGAGG	RP to obtain the *eshA* probe for EMSA
**actII-orf4_F**	TCACCTCGACCGGTGGCGTG	FP to obtain the *actII-orf4* probe for EMSA
**actII-orf4_R**	GAAGGGTCCTCGCGCCGG	RP to obtain the *actII-orf*4 probe for EMSA
**gvpZ2_F**	GACGCACATCATGGAGACCGTC	FP to obtain the *gvpZ2* probe for EMSA
**gvpZ2_R**	CTTCCAGCTGCGGGTCGA	RP to obtain the *gvpZ2* probe for EMSA
**cpkA_F**	CCCGGCTGCTACGGAATTGTT	FP to obtain the *cpkA* probe for EMSA
**cpkA_R**	CAACGGGCCGGTCATCAG	RP to obtain the *cpkA* probe for EMSA
**PHO-47**	ATGGGTCCTCCAGGGTTCGA	FP to obtain the SCO0255 probe for EMSA
**PHO-48**	CGCTGCCAGGAGTTGAGGAT	RP to obtain the SCO0255 probe for EMSA
**PHO-53**	GGACGATCGATTTGCAGGTC	FP to obtain the *ectA* probe for EMSA
**PHO-54**	CGGATACTCGCACAAGCAGA	RP to obtain the *ectA* probe for EMSA

1) Forward and reverse primers are abbreviated as FP and RP, respectively
2) The oligonucleotides used for the SCO0255 and *ectA* probe amplification have been already described by Sola-Landa et al. (2013)
