# Supplementary material for: ArgR of Streptomyces coelicolor Is a Pleiotropic Transcriptional Regulator: Effect on the Transcriptome, Antibiotic Production, and Differentiation in Liquid Cultures
Source: Front Microbiol. 2018 Mar 1;9:361. doi: 10.3389/fmicb.2018.00361 (PMC5839063; doi:10.3389/fmicb.2018.00361)
Supplement: Supplementary file 6 [file Image1.PDF]

Figure S1. Quantification of the sporulation process.

Master images used to quantify the sporulation-like processes in the DargR mutant. Segmented hyphae are labelled as “1”; non-segmented hyphae are labelled as “2”. Segments used for length quantification are marked.

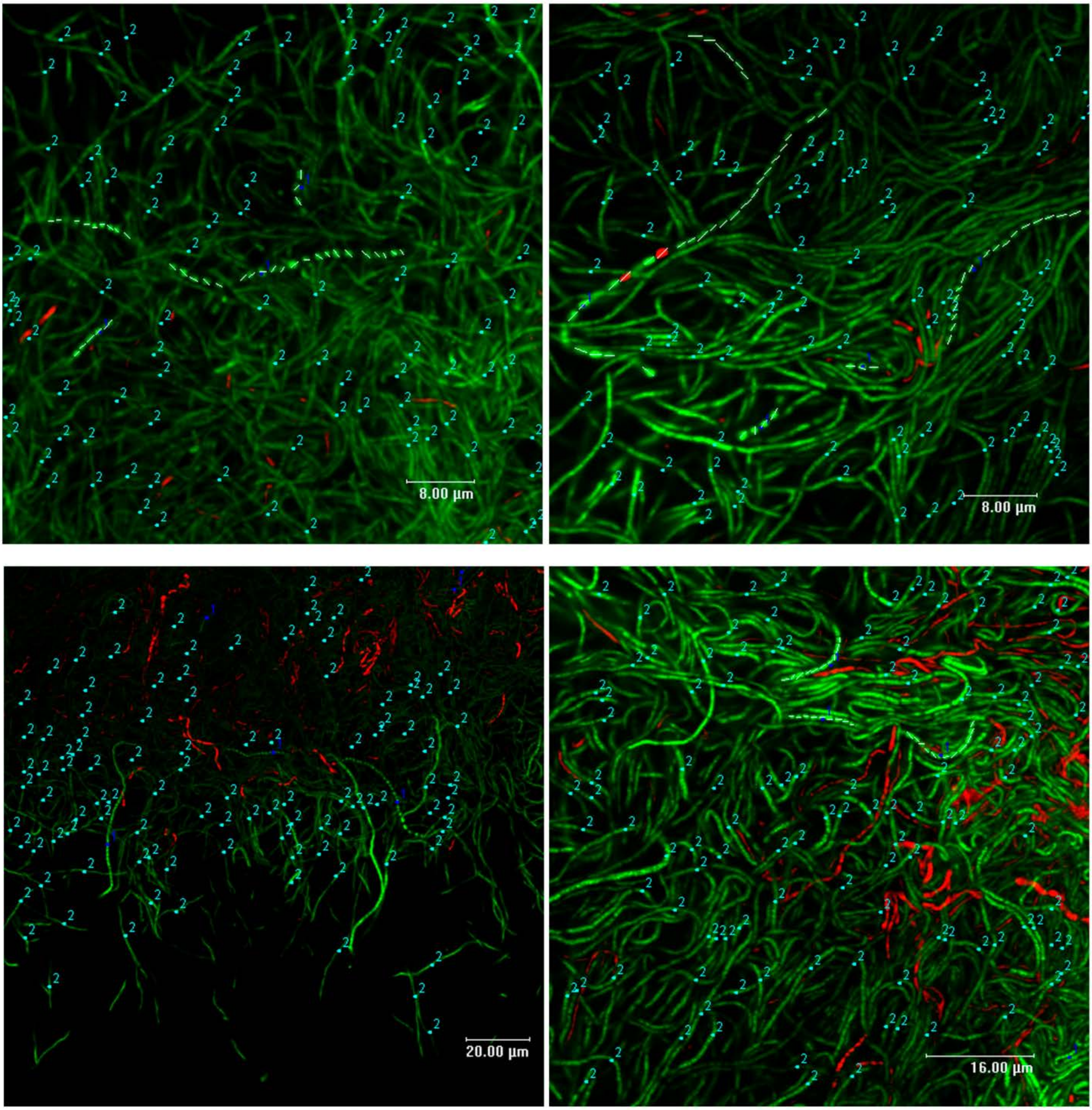

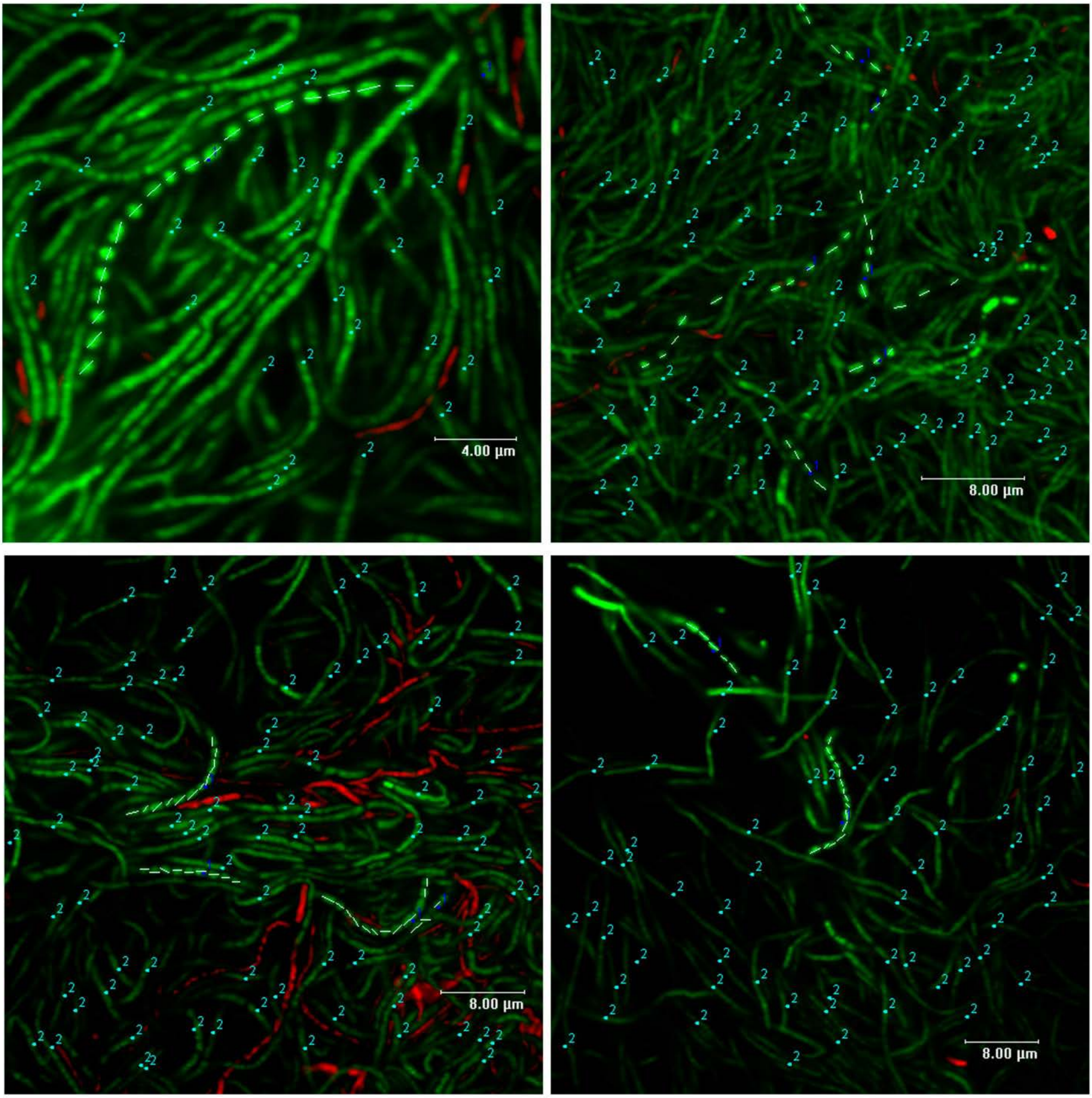
4.00 µm
8.00 µm
8.00 µm
8.00 µm
