# Supplementary material for: ArgR of Streptomyces coelicolor Is a Pleiotropic Transcriptional Regulator: Effect on the Transcriptome, Antibiotic Production, and Differentiation in Liquid Cultures
Source: Front Microbiol. 2018 Mar 1;9:361. doi: 10.3389/fmicb.2018.00361 (PMC5839063; doi:10.3389/fmicb.2018.00361)
Supplement: Supplementary file 7 [file Image2.PDF]

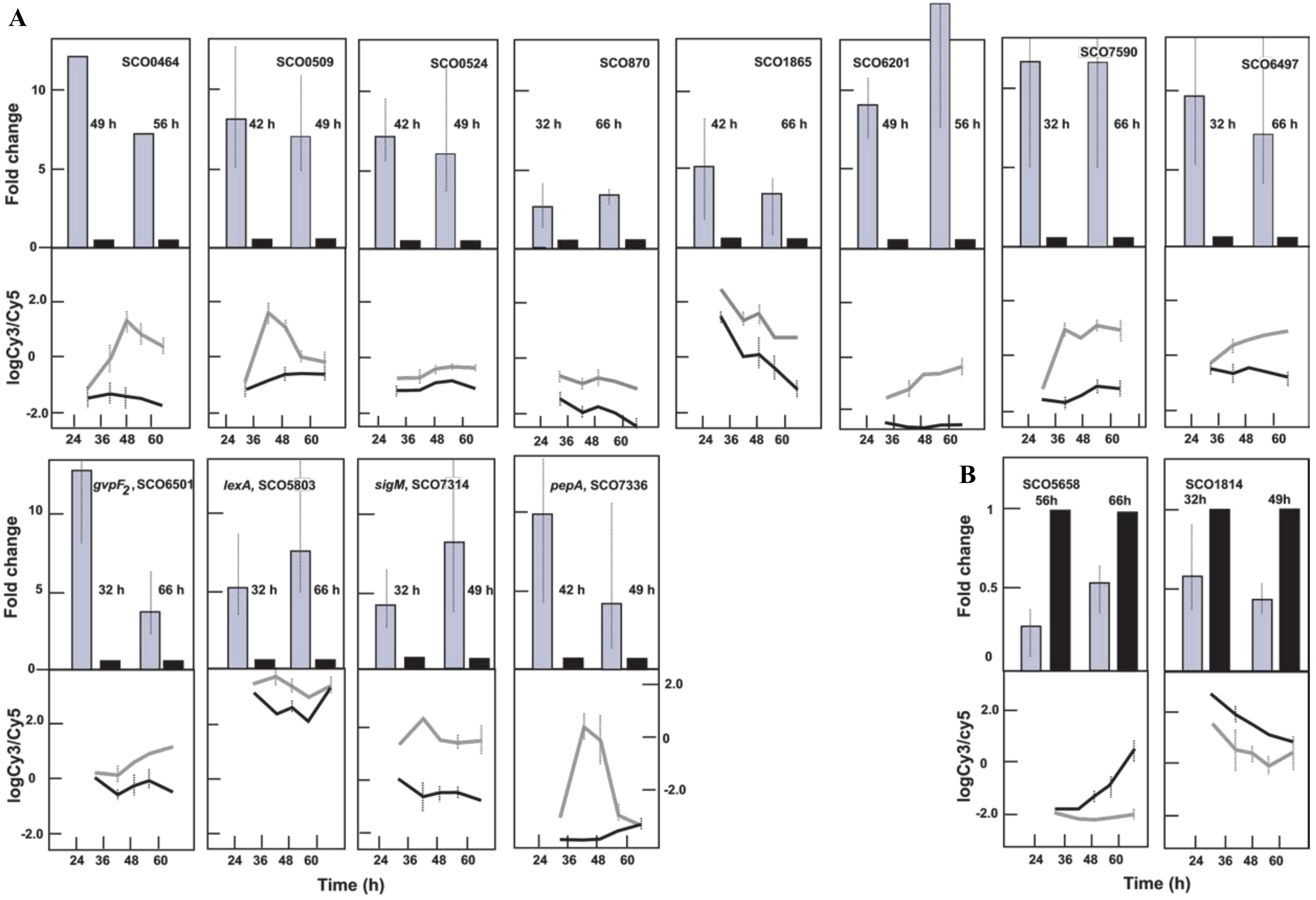

Figure S2. Validation by qRT-PCR of the microarray data corresponding to genes showing different expression profile. A) Validation of genes overexpressed in the *argR* mutant. B) Validation of genes underexpressed in the *argR* mutant. The expression is presented in relation to the control strain taken as 1. In the upper panels is indicated the name of the gene and the time of culture at which the qRT-PCR was performed. In the corresponding lower panel is shown the profile of the gene in the microarray study. Black bars and black lines correspond to the control strain *S. coelicolor* M145. Gray bars and gray lanes correspond to *S. coelicolor argR*.
