# Supplementary material for: ArgR of Streptomyces coelicolor Is a Pleiotropic Transcriptional Regulator: Effect on the Transcriptome, Antibiotic Production, and Differentiation in Liquid Cultures
Source: Front Microbiol. 2018 Mar 1;9:361. doi: 10.3389/fmicb.2018.00361 (PMC5839063; doi:10.3389/fmicb.2018.00361)
Supplement: Supplementary file 8 [file Presentation1.PDF]

Supplementary Material. Frontiers in Microbiology.

ArgR of *Streptomyces coelicolor* is a pleiotropic transcriptional regulator: Effect on the transcriptome, antibiotic production, and differentiation in liquid cultures

Alma Botas[1, 2], Rosario Pérez-Redondo[2], Antonio Rodríguez-García[1, 2], Ruben Álvarez-Álvarez[1], Paula Yagüe[3], Angel Manteca[3] and Paloma Liras[1].

1) Área de Microbiología, Facultad de Ciencias Biológicas y Ambientales, Universidad de León, 24071 León, Spain
2) Instituto de Biotecnología de León, INBIOTEC, Avda. Real nº1, 24006 León, Spain
3) Área de Microbiología, Departamento de Biología Funcional e IUOPA, Facultad de Medicina, Universidad de Oviedo, 33006 Oviedo, Spain
